# The Reduction of Anastomosis-Related Morbidity Using the Kono-S Anastomosis in Patients with Crohn’s Disease: A Meta-Analysis

**DOI:** 10.3390/jcm13092461

**Published:** 2024-04-23

**Authors:** Ioannis Baloyiannis, Konstantinos Perivoliotis, Chamaidi Sarakatsianou, Charito Chatzinikolaou, George Tzovaras

**Affiliations:** 1 Department of Surgery, University Hospital of Larissa, 41110 Larissa, Greece; balioan@hotmail.com (I.B.); heidisarak@gmail.com (C.S.); geotzovaras@gmail.com (G.T.); 2 Department of Surgery, General Hospital of Volos, 38222 Volos, Greece; xaritoxatz@gmail.com

**Keywords:** Kono-S, Crohn, anastomosis, complications, morbidity, meta-analysis

## Abstract

**(1) Background**: we conducted this study to evaluate the effect of Kono-S anastomosis on postoperative morbidity after bowel resection for Crohn’s disease. **(2) Methods**: This study adhered to the PRISMA guidelines and the Cochrane Handbook for Systematic Reviews of Interventions. The primary endpoint was the overall complications rate. Secondary outcomes included specific complications analyses, disease recurrence and efficiency endpoints. A systematic literature screening was performed in major electronic scholar databases (Medline, Scopus, Web of Science), from inception to 17 January 2024. Both Random (RE) and Fixed Effects (FE) models were estimated; the reported analysis was based on the Cochran Q test results. **(3) Results**: Overall, eight studies and 913 patients were included in this meta-analysis. Pooled analyses confirmed that Kono-S was not superior in terms of overall morbidity (OR: 0.69 [0.42, 1.15], *p* = 0.16). Kono-S displayed a reduced risk for anastomotic leakage (OR: 0.34 [0.16, 0.71], *p* = 0.004) and reoperation (OR: 0.12 [0.05, 0.27], *p* < 0.001), and a shortened length of hospital stay (WMD: −0.54 [−0.73, −0.34], *p* < 0.001). On the contrary, Kono-S results in higher rates of postoperative SSIs (OR: 1.85 [1.02, 3.35], *p* = 0.04). **(4) Conclusions**: This study confirms a comparable morbidity, but a lower risk of anastomotic leak and reoperation of Kono-S over conventional anastomoses. Further high quality studies are required to validate these findings.

## 1. Introduction

### 1.1. Rationale

Crohn’s disease (CD) is a chronic idiopathic disorder that is characterized of transmural inflammation of the gastrointestinal tract, alongside extraintestinal manifestations [1]. Current epidemiologic studies suggest that CD has an increasing prevalence, especially in industrialized countries, with a peaked incidence in young adults [2,3].

Despite recent advances in overall management, CD has a detrimental impact on patients’ health-related quality of life due to work disability, disease relapse, treatment side-effects and repeated hospitalizations [4]. In addition to these, in most cases, the administration of immunosuppressors and biologic therapies fails to control the natural course of the disease [5,6]; indeed, almost 80% of CD patients will ultimately be submitted to bowel resection [3]. However, removing the affected bowel segment is not curative and, due to 50% clinical recurrence rates, many patients will require multiple resections [3,7].

The anastomotic site is of pivotal importance, and almost 90% of patients will have an endoscopic recurrence at 3 years postoperatively [6]. Several risk factors have been identified as early anastomotic recurrence predictors, including smoking, disease behavior, perianal involvement, prior resections, histologic characteristics, patient demographics, disease location, and postoperative complications [8,9,10,11]. Moreover, the optimal anastomotic technique has been a matter of debate, with handsewn end-to-end and stapled side-to-side configurations being the most frequently performed techniques [12]. Previous pooled analyses reported conflicting results regarding anastomotic leakage, morbidity, hospitalization duration, and recurrence risk between the two anastomotic approaches [13,14].

In 2011, Kono et al. [15] described a novel anastomotic technique after bowel resection for CD, acknowledging the role of mesenteric inflammation and attempting to prevent early disease recurrence. In this technique, the mesentery is excluded through the construction of supporting columns [16]. The latter allows the orientation of the anastomosis to be maintained, and secures a wide lumen [6,15,17]. Kono-S is completed by the performance of an antimesenteric handsewn anastomosis in a single-layer Gambee manner with 3/0 Vicryl running sutures [6,15,17].

The initial report by Kono et al. [15] suggested a significantly lower endoscopic recurrence score at 5 years postoperative, with no increase in postoperative morbidity. Subsequent trials, though, failed to confirm this superiority of Kono-S over conventional anastomoses [16,18,19]. On the contrary, a recent pooled analysis [20] reported a 24.7% incidence of endoscopic recurrence in the Kono-S arm, compared to the respective 42.6% in the comparison group.

However, current evidence regarding the effect of the novel anastomotic technique on perioperative morbidity is still inconclusive [16,18,19]. More specifically, a meta-analysis by Ng et al. [12] estimated a significantly lower risk (1%) of anastomotic leak when Kono-S was performed. Similarly, Shimada et al. [6] reported that Kono-S resulted in a significant reduction in anastomotic leakage rates, while Kelm et al. [18] associated the new approach with a higher risk of surgical site infections. In addition to these, recently published comparative studies [17,19] provided contradictive results regarding the comparability of Kono-S and conventional anastomotic techniques in terms of postoperative complications. Therefore, the need for updated evidence and ranking of the two approaches considering morbidity and perioperative efficacy, is thoroughly justified.

### 1.2. Objectives

Taking into consideration the above-mentioned evidence, we designed and conducted the present meta-analysis to evaluate the role of the Kono-S anastomosis in postoperative morbidity and efficiency after bowel resection for CD.

## 2. Materials and Methods

### 2.1. Study Protocol

This meta-analysis was conducted according to the PRISMA guidelines [21] and the Cochrane Handbook for Systematic Reviews of Interventions [22]. The review protocol was not pre-registered.

### 2.2. Endpoints

The primary endpoint of this study was the comparison of Kono-S and conventional (CONV) anastomosis regarding the overall complications rate, in patients submitted to surgical resection for CD. Secondary outcomes included specific complication analyses (Clavien–Dindo ≥ III, intraabdominal abscess, surgical site infection-SSI, ileus, bleeding, anastomotic leakage, readmission, and reoperation), disease recurrence (clinical recurrence and Rutgeerts score > i2 [23]), and efficiency endpoints (operation duration and length of hospital stay (LOS)). Conventional anastomosis was considered any standardized anastomotic technique, besides Kono-S, regardless of its layout (end-to-side, end-to-end, side-to-side) and technique (handsewn, stapled).

### 2.3. Eligibility Criteria

All clinical studies that compared the two anastomotic techniques after any bowel resection, in patients with CD, whose data were extractable, and the original report was written in English were considered as eligible. The following exclusion criteria were applied: (1) non-human studies, (2) no outcomes of interest, (3) no comparison group, (4) article not written in English, (5) irretrievable data, and (6) manuscripts in the form of editorials, case reports, expert opinions, or conference abstracts. There was no restriction in terms of bowel resection type.

### 2.4. Literature Search

After the removal of duplicate entries, the titles and abstracts of the search results were screened based on the eligibility criteria. Consequently, a full text evaluation of the remaining manuscripts was performed. All literature searches, data extractions, and quality assessments were performed in duplicate and blindly by two independent researchers (P.K. and B.I.). In case of a discrepancy that was not resolved by mutual revision, the opinion of a third investigator was considered (T.G.).

Methodological assessment was based on the ROBINS-I [24] and RoB 2 tool [25] (Website: https://www.riskofbias.info/, access on date: 21 January 2024) for non-randomized and randomized controlled trials (RCTs), respectively. Interrater agreement was estimated through the calculation of Cohen’s k statistic.

### 2.5. Study Selection and Data Collection

To identify eligible studies, a systematic literature screening was performed in major electronic scholar databases (Medline, Scopus, Web of Science) from inception to 17 January 2024. The following keywords were introduced as search terms: “Kono-S”. To avoid missing any study, a broad search strategy was introduced, with minimum restrictive terms.

After the identification of the eligible studies the data extraction process was initiated. Besides the analyzed endpoints, the following data were recorded: included studies’ characteristics (first author, country, study type, number of centers, publication year, study period, sample per arm, gender, age and Body Mass Index (BMI) allocation, follow-up period), patient characteristics (American Society of Anesthesiologists (ASA) score, smoking, previous operations, perianal disease and Vienna classification), previous treatment characteristics (type of medications), and surgical approach characteristics (previous surgical experience, number of surgeons, emergency operations, resection site, type of approach, anastomotic technique, length of resected bowel).

### 2.6. Statistical Analysis

Statistical analyses were performed in Cochrane Collaboration RevMan (Version 5.4.1 Copenhagen: The Cochrane Collaboration, 2020) and IBM SPSS Statistics for Windows (Version 29.0.2.0 Armonk, NY: IBM Corp). Categorical and continuous endpoints were reported as odds ratio (OR) and weighted mean difference (WMD), respectively. All variables were provided with the corresponding 95% confidence interval (95%CI).

In cases where the mean or the standard deviation (SD) of a variable was not reported, they were estimated from the respective median, range, or interquartile range (IQR), based on the formula described by Hozo et al. [26]. Meta-analysis estimations utilized the Mantel–Haenszel (MH) and inverse variance (IV) algorithms. Heterogeneity estimation included the calculation of I^2^. Both random -RE and fixed effects -FE models were estimated; the reported analysis was based on the Cochran Q test results (Q *p* < 0.1). Explanatory analyses included subgroup analysis and meta-regression. Meta-regression was based on the RE model and utilized a DerSimonian–Laird estimator. Statistical significance was considered at the level of *p* < 0.05.

### 2.7. Risk of Bias across Studies

The funnel plot of all outcomes was visually evaluated for the presence of publication bias.

## 3. Results

**Figure 1:**
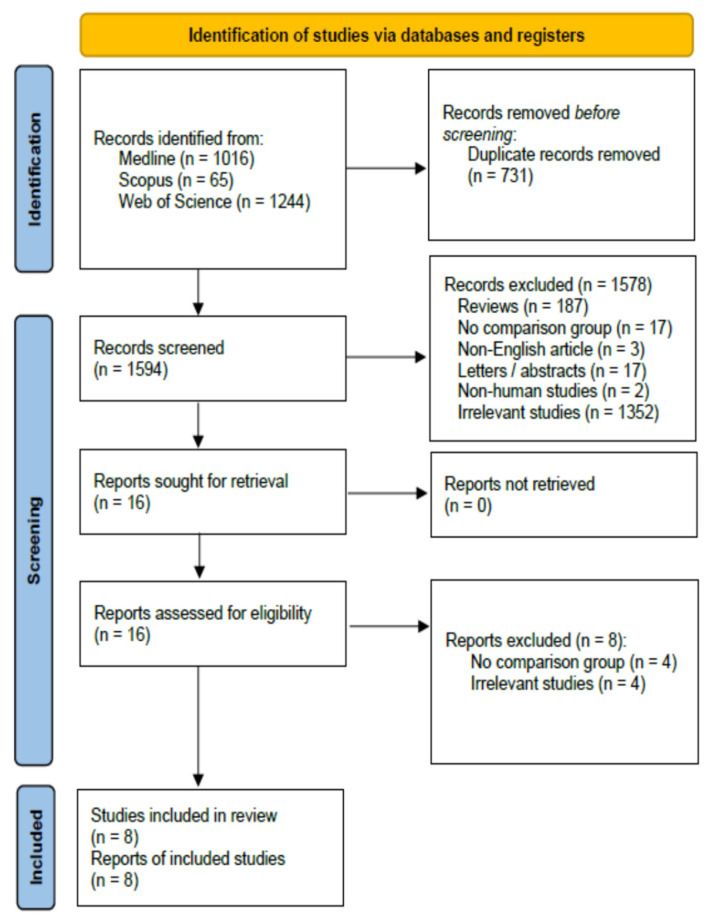
PRISMA study flow diagram. For more information, visit: https://www.prisma-stataement.org (accessed on 18 January 2024).

The application of the screening algorithm resulted to the retrieval of 2325 entries (Figure 1). After the removal of 731 duplicates, the titles and abstracts of the remaining articles were reviewed. Overall, 1578 records (reviews and meta-analyses: 187; single armed study: 17; non-English article: 3; letters, expert opinions, or conference abstracts: 17; experimental studies: 2; irrelevant records: 1352) were excluded during this step. Full text assessment identified four studies with no comparison group and four irrelevant articles. Consequently, eight studies [5,6,15,17,18,19,27] were included in the qualitative and quantitative synthesis.

Overall, 913 patients were included in this meta-analysis (Table 1). There was only one RCT [5]; the remaining trials applied either a prospective or a retrospective methodology. Most studies were performed in a single center [5,6,16,17,18,27], and the publication period spanned from 2011 to 2023. Data regarding gender, age, and BMI allocation are also provided in Table 1. Mean follow up spanned from 6.55 to 89 months.

Data regarding the ASA status of the patients were provided only in three [16,17,19] studies (Supplementary Materials). Overall, 141 patients had received a previous operation. In total, 71 patients displayed perianal disease manifestations. Furthermore, 89, 279 and 242 CD cases were classified as inflammatory (B1), stricturing (B2) and penetrating (B3) disease behavior, respectively. Biologics were administered in 403 patients.

Overall, three studies [17,18,27] confirmed previous surgical expertise (Supplementary Materials Tables S1–S5). Similarly, data regarding the number of operating surgeons were scarce. Only 19 resections were performed in an emergency setting. All studies, except two [6,15], reported data on ileocolic anastomoses (Supplementary Materials). Most operations were performed in a laparoscopic approach. Open conversion was required in 22 cases. Finally, 162 stapled conventional anastomoses were included in the comparative analyses.

Quality assessment of the eligible studies highlighted moderate to serious methodological deficits in most non-RCTs. The RCT by Luglio et al. [5] was graded as having some concerns regarding the overall risk of bias. There was an adequate level of agreement in both tools (RoB 2 Cohen k statistic:1 *p* = 0.025, ROBINS-I Cohen k statistic: 0.85 *p* < 0.001).

**Figure 2:**
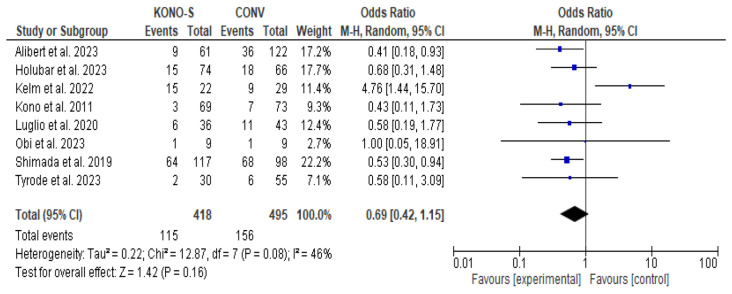
Overall complications forest plot [5,6,15,16,17,18,19,27].

All eligible studies provided data regarding the primary outcome (Figure 2, Table 2). Pooled evidence did not confirm a superiority (OR: 0.69 [0.42, 1.15], *p* = 0.16) of Kono-S over conventional anastomosis after bowel resection in patients with CD. Due to significant heterogeneity levels (I^2^: 46%, *p* = 0.08), further explanatory analyses were performed. The results of meta-regression (Supplementary Materials) could not confirm a significant effect of any analyzed variable (publication year, sample size, gender, age, BMI, follow up, smoking, previous operation, perianal disease, anti-TNF medication, laparoscopic approach, stapled conventional anastomosis, and resected length of bowel). Stratifying for ileocolic anastomoses (OR: 0.83 [0.4, 1.73], *p* = 0.61) and side-to-side conventional anastomoses only (OR: 0.89 [0.29, 2.75], *p* = 0.84) did not alter the overall outcome. A non-significant result was also estimated in the experienced surgeons’ subgroup (OR: 1.67 [0.37, 7.64], *p* = 0.51). A significant superiority of Kono-S (Supplementary Materials) was confirmed in the prospective (OR: 0.47 [0.24, 0.89], *p* = 0.02), but not in the retrospective studies subgroup. Exclusion of high risk of bias studies resulted in a significant effect of the experimental technique (OR: 0.47 [0.24, 0.89], *p* = 0.02).

In terms of secondary outcomes (Table 2, Supplementary Materials), Kono-S displayed comparable rates of Clavien–Dindo ≥ III complications (*p* = 0.18), intraabdominal abscesses (*p* = 0.18), postoperative ileus (*p* = 0.87) and bleeding (*p* = 0.1). Similarly, there was no difference in terms of readmission rates (*p* = 0.21). On the contrary, Kono-S was associated with a significantly higher risk of SSIs (OR: 1.85 [1.02, 3.35], *p* = 0.04). Anastomotic leakage (OR: 0.34 [0.16, 0.71], *p* = 0.004) and reoperation rates (OR: 0.12 [0.05, 0.27], *p* < 0.001) were significantly decreased when Kono-S was applied.

Pooled analyses could not confirm an improvement in clinical (*p* = 0.12) or endoscopic recurrence (*p* = 0.22) with Kono-S. Furthermore, the introduction of Kono-S as an anastomotic technique did not alter the procedure duration (*p* = 0.29); however, it resulted in a significant reduction in LOS by a mean of 0.54 days (WMD: −0.54 [−0.73, −0.34], *p* < 0.001).

Inspection of the primary endpoint funnel plot (Supplementary Materials) showed a symmetrical distribution of the studies over the combined effect size line. The funnel plots of the secondary outcomes are also provided in Supplementary Materials. 

## 4. Discussion

### 4.1. Summary of Evidence

Our study is an effort to provide updated evidence regarding the efficacy of the novel Kono-S anastomotic technique in reducing postoperative morbidity and recurrence rates after bowel resection in CD patients. Our results suggest that Kono-S is not effective in minimizing clinical and endoscopic recurrence, and does not provide an enhanced overall safety profile. Despite these, a lower risk of anastomotic leakage and reoperation was confirmed.

Optimal therapeutic management of CD is based on disease staging, patient risk stratification and preferences, and clinical characteristics [28]. In most cases, the initial approach includes the administration of steroids to alleviate the symptoms and the subsequent introduction of biologics to control and reduce the risk of disease flares [28]. A significant proportion of patients, though, will ultimately undergo bowel resection due to the development of stenoses, fistulas or disease refractory to conventional immunomodulatory therapy [28]. Additionally, in specific disease phenotypes, including inflammatory ileocolic CD, upfront surgery is considered as a valid option due to optimal results [3,29,30]. In the LIR!C RCT [31], laparoscopic ileocecal resection was compared to infliximab for limited non-stricturing ileocecal CD, with comparable results in terms of restoring quality of life and overall morbidity. A significant finding of this study was that one-third of patients in the biologic group required resection compared to one fourth of the surgical arm that received infliximab [31].

Consequently, it became apparent that, to enhance postoperative outcomes, the surgical approach, including the anastomotic technique, should be optimized. In the CAST trial [32], side-to-side was compared to end-to-end anastomosis after ileocecal resection; no significant difference in the endoscopic and symptomatic recurrence rates was found during follow-up. Comparability of the two techniques in early postoperative outcomes was also reported by the ISRCTN-45665492 study [33]. Contrary to these, a recent network meta-analysis [34] suggested the superiority of side-to-side anastomosis in terms of overall morbidity, clinical recurrence, and reoperation. Furthermore, the role of mesenteric resection in disease outcome has also attracted the interest of researchers [35]. As an answer to these, Kono et al. [15] described the homonymous anastomotic layout with specific technical features, and also reported promising postoperative results.

Despite being a side-to-side handsewn anastomosis, Kono-S incorporates several technical features that render it a complex procedural step [18]. As such, to minimize postoperative morbidity, structured training should be considered prior to performing Kono-S [18]. More specifically, based on recent studies, approximately 20 cases are required to overcome the learning curve of this new technique [18]. This further highlights the need to properly assess the safety of this new approach, prior to extensively applying it to CD patients. In a recent cohort by Tyrode et al. [17], Kono-S did not have a higher morbidity profile compared to conventional handsewn, side-to-side anastomoses. This was also confirmed in a meta-analysis by Ng et al. [12], where minimal rates of complications were reported. Our pooled analyses calculated a 27.5% and 31.5% overall morbidity rate for Kono-S and a control group, respectively. This was reduced to 3.7% and 6.6% when assessing severe (Clavien–Dindo ≥ III) complications. Statistical significance was not reached in any of these comparisons, and the influencing factors were not identified.

Previous experimental and clinical studies confirmed that the bowel segments affected by CD have an over 50% decrease in blood flow [15]. The imaging quantification of this clinical parameter is currently under evaluation with novel techniques, including shear wave and strain elastography [36]. Due to its configuration, the novel anastomotic technique presents several advantages, including the preservation of bowel perfusion and innervation, factors directly associated with optimal anastomotic healing [15]. Initial reports did not suggest a superiority of Kono-S over conventional techniques in terms of anastomotic healing [15]. In a large cohort by Shimada et al. [6], Kono-S displayed a lower rate (5.1% vs. 17.3%) of anastomotic leakage when compared to layer-to-layer end-to-end anastomosis; this, though, did not reach statistical significance. Interestingly, our pooled estimations suggested a significant difference in favor of Kono-S regarding the risk of postoperative anastomotic leakage.

Our meta-analysis confirmed a higher rate of SSIs in patients submitted to Kono-S anastomosis after bowel resection. In addition to this, a low heterogeneity level was identified. Although no single study had a significant effect size, the trials of Kelm et al. [18] and Shimada et al. [6] reported a higher incidence of infections in the experimental group. As mentioned by Kelm et al. [18], a possible explanation for this could be the access routes used for the anastomoses after different approaches (suprapubic for conventional and periumbilical for Kono-S).

The mesentery in CD develops characteristic macroscopic and microscopic changes, including fat thickening and hyperplasia of adipocytes and connective tissue [19]. Several researchers suggested that the mesentery has a pivotal role in the pathogenesis and recurrence of the disease, given the fact that mucosal inflammation is usually located in the mesenteric side and has a positive association with the mesenteric inflammation [19]. To prevent this, Kono et al. [15] proposed the creation of a supporting column and the formation of the anastomosis on the antimesenteric axis [15]. Besides the biomechanistic properties, Kono-S may also prevent early recurrence through the preservation of gut flora [12]; the isoperistaltic layout and the supporting column minimizes microbiome changes, especially in cases of ileocecal resections [12].

The potential of Kono-S in reducing recurrence rates has been extensively evaluated in multiple clinical scenarios [12]. Comparing Kono-S to conventional anastomoses, Kono et al. [15] found that, although 1-year endoscopic recurrence rates were comparable, the experimental group displayed a lower mean Rutgeert score at 5 years. In the SuPREMe-CD RCT [5], Kono-S was compared to a conventional side-to-side stapled anastomosis and had a lower risk for endoscopic recurrence at 6 and 18 months postoperatively. Logistic regression also confirmed the anastomotic technique as the sole predictor of endoscopic recurrence and a lower recurrence-free survival was reported in the conventional study arm [5]. On the contrary, in the propensity score matched cohort of the KoCoRICCO study [19], Kono-S was not associated with lower endoscopic recurrence events. In our study, the two approaches did not differ in terms of clinical and endoscopic recurrence (Rutgeert score ≥ i2). However, when evaluating such outcomes, several factors should be acknowledged, including time endpoints, disease behavior, definition of recurrence and control technique. Regarding the latter, it has been confirmed that the mechanical staple line may falsely be diagnosed with endoscopic recurrence, whereas handsewn anastomoses heal without ulcerations [19].

In the Kono-S technique, a complex handsewn anastomosis is performed; thus, theoretically, prolonging the operative time [16]. Indeed, previous studies suggested that due to the additional hand-sewing, Kono-S resulted to an average 30 min longer operation duration [16]. In the previous meta-analysis by Ng et al. [12], the mean operative time of resection and restoration of bowel continuity was 179 min, which was comparable to the respective control group. Our results were like the latter, since no significant difference was found between the Kono-S and conventional anastomosis groups. It must be noted, though, that this estimation was plagued by high heterogeneity levels; thus, suggesting that factors like the Kono-S learning curve status and the conventional technique that was used as comparator could have influenced the results.

We confirmed that patients submitted to resection and restoration of bowel continuity with this novel technique, were discharged earlier compared to the conventional group. Previous cohorts, though, did not identify a hospitalization duration benefit of Kono-S [5,16]. Moreover, data regarding postoperative bowel function and patient mobilization were scarce and, therefore, no analysis of other recovery endpoints was available. Therefore, this significant effect could be associated with the previously reported lower rates of anastomotic complications and reoperation in the Kono-S group.

### 4.2. Limitations

Prior to the appraisal of the results of this study, several limitations should be acknowledged. First, most of the eligible studies did not include an adequate randomization or blinding algorithm, thus reducing the validity of the pooled analyses. Furthermore, the quality assessment highlighted several methodological deficits that could have contributed to the overall bias. Moreover, the small sample size in the included trials reduced the power of the pooled statistical calculations. Additionally, the inherent heterogeneity in terms of patient characteristics, disease stage, perioperative treatment regimens, and the conventional anastomotic technique, further impacted the significance of the estimate endpoints. Finally, the divergence in the reported follow-up period could have affected several time-related endpoints including morbidity and disease recurrence.

## 5. Conclusions

This meta-analysis failed to estimate a significant effect of Kono-S over conventional anastomoses, in reducing the overall postoperative morbidity rates, after bowel resection for CD. Kono-S displayed a reduced risk for anastomotic leakage and reoperation, and a shortened length of hospital stay. On the contrary, Kono-S resulted in higher rates of postoperative SSIs. Due to several study limitations, further higher quality RCTs are required to delineate the exact role of Kono-S in patients submitted to surgery for Crohn’s disease.

## Figures and Tables

**Table 1:** Included studies.

Author	Country	Study Type	Center	Year	Study Period	Group	Sample	Gender (M)	Age	BMI	Follow Up
Alibert et al. [19]	France	prospective	multi	2023	2020–2022	KONO-S	61	26	37 (4.75)	21.9 (1.17)	6.7 (0.41)
						CONV	122	55	34 (3)	20.9 (0.90)	
Holubar et al. [16]	USA	retrospective	single	2023	2015–2022	KONO-S	74	36	38.2 (16.3)	25.1 (5.6)	n/a
						CONV	66	33	37.9 (15.5)	25.4 (5.6)	
Obi et al. [27]	USA	retrospective	single	2023	2019–2022	KONO-S	9	4	15.4	21.6	6.55
						CONV	9	3	16.2	20	7.57
Tyrode et al. [17]	France	retrospective	single	2023	2020–2022	KONO-S	30	13	32.2 (13.4)	22.3 (3.92)	12
						CONV	55	24	36.1 (15.6)	23.5 (6)	
Kelm et al. [18]	Germany	retrospective	single	2022	2019–2021	KONO-S	22	14	37.4 (10.5)	24.3	8.8 (2.5)
						CONV	29	14	36.8 (13.7)	22.8	
Luglio et al. [5]	Italy	RCT	single	2020	2015–2017	KONO-S	36	18	34 (6.25)	n/a	24
						CONV	43	22	43 (8.25)		
Shimada et al. [6]	Japan	retrospective	single	2019	2006–2016	KONO-S	117	84	39 (11.8)	18.9 (2.51)	38 (23.7)
						CONV	98	74	34 (11.1)	18.6 (2.44)	89 (34)
Kono et al. [15]	Japan	retrospective	multi	2011	2003–2009	KONO-S	69	57	31 (10.7)	n/a	42 (18.7)
						CONV	73	58	28 (12)		52 (29.7)

n/a: not available.

**Table 2:** Primary and secondary outcomes.

Outcome	Studies	Participants	Statistical Method	Effect Estimate 95%CI	*p*	I^2^	Heterogeneity *p*
Overall Complications	8	913	Random Effects	0.69 [0.42, 1.15]	0.16	46%	0.08
CD ≥ III	4	459	Fixed Effects	0.54 [0.22, 1.32]	0.18	14%	0.32
Intrabdominal Abscess	6	624	Fixed Effects	0.62 [0.31, 1.25]	0.18	0%	0.68
SSI	7	730	Fixed Effects	1.85 [1.02, 3.35]	0.04	0%	0.71
Ileus	6	645	Fixed Effects	0.95 [0.55, 1.66]	0.87	0%	0.88
Bleeding	4	446	Fixed Effects	0.34 [0.09, 1.25]	0.1	0%	0.82
Leakage	7	828	Fixed Effects	0.34 [0.16, 0.71]	0.004	0%	0.66
Readmission	4	453	Fixed Effects	0.59 [0.26, 1.35]	0.21	5%	0.37
Reoperation	4	515	Fixed Effects	0.12 [0.05, 0.27]	<0.001	0%	0.66
>i2	5	540	Random Effects	0.66 [0.33, 1.29]	0.22	68%	0.01
Clinical Recurrence	4	562	Random Effects	0.42 [0.14, 1.24]	0.12	75%	0.007
Operation Duration [minutes]	5	702	Random Effects	5.71 [−4.93, 16.36]	0.29	83%	<0.001
LOS [days]	4	487	Fixed Effects	−0.54 [−0.73, −0.34]	<0.001	0%	0.48

## Data Availability

Data sharing not applicable to this article as no datasets were generated or analyzed during the current study.

## Supplementary Materials

The following supporting information can be downloaded at: https://www.mdpi.com/article/10.3390/jcm13092461/s1, Table S1. Included patient characteristics; Table S2. Previous treatment characteristics; Table S3. Surgical approach characteristics; Table S4. Overall Morbidity Meta-Regression; Table S5. Subgroup Analysis Based on Study Type; Figure S1. Risk of Bias 2 traffic light plot; Figure S2. ROBINS-I traffic light plot; Figure S3. CD>III forest plot; Figure S4. Intraabdominal abscess forest plot; Figure S5. SSI forest plot; Figure S6. Ileus forest plot; Figure S7. Bleeding forest plot; Figure S8. Leakage forest plot; Figure S9. Readmission forest plot; Figure S10. Reoperation forest plot; Figure S11. >i2 forest plot; Figure S12. Clinical recurrence forest plot; Figure S13. Operation duration forest plot; Figure S14. LOS forest plot; Figure S15. Overall complications funnel plot; Figure S16. CD>III funnel plot; Figure S17. Intraabdominal abscess funnel plot; Figure S18. SSI funnel plot; Figure S19. Ileus funnel plot; Figure S20. Bleeding funnel plot; Figure S21. Leakage funnel plot; Figure S22. Readmission funnel plot; Figure S23. Reoperation funnel plot; Figure S24. >i2 funnel plot; Figure S25. Clinical recurrence funnel plot; Figure S26. Operation duration funnel plot; Figure S27. LOS funnel plot.
