# Supplementary material for: The Reduction of Anastomosis-Related Morbidity Using the Kono-S Anastomosis in Patients with Crohn’s Disease: A Meta-Analysis
Source: J Clin Med. 2024 Apr 23;13(9):2461. doi: 10.3390/jcm13092461 (PMC11084922; doi:10.3390/jcm13092461)
Supplement: Supplementary file 1 [file jcm-13-02461-s001.zip › jcm-2907491-supplementary.pdf]

Author	Group	Sample	ASA				Smoking	Previous Operation	Perianal Disease	Vienna Classification						
			**I**	**II**	**III**	**IV**				**A1**	**A2**	**L1**	**L3**	**B1**	**B2**	**B3**
Alibert et al.	KONO-S	61	59		2	0	16	12	13	n/a	n/a	n/a	n/a	24	47	24
	CONV	122	113		4	0	26	26	20					44	80	49
Holubar et al.	KONO-S	74	n/a		27		12	n/a	7	67	7	23	51	7	49	18
	CONV	66			33		11		3	65	1	25	21	10	33	18
Obi et al.	KONO-S	9	n/a				n/a	n/a	n/a	n/a						
	CONV	9														
Tyrode et al.	KONO-S	30	10	19	1	0	8	n/a	8	n/a						15
	CONV	55	13	39	3	0	20		20							30
Kelm et al.	KONO-S	22	n/a				5	6	n/a	n/a				1	17	4
	CONV	29					11	6						3	22	4
Luglio et al.	KONO-S	36	n/a				11	19	n/a	n/a					15	16
	CONV	43					10	28							16	19
Shimada et al.	KONO-S	117	n/a				7	n/a	n/a	n/a						
	CONV	98					4									
Kono et al.	KONO-S	69	n/a				n/a	21	n/a	n/a						22
	CONV	73						23								23

Table S1. Included patient characteristics.

Author	Group	Sample	Previous Treatment							
			**Mesalazine**	**Budesonide**	**Thiopurin**	**Azathioprine**	**Methotrexate**	**Anti TNF**	**Ustekinumab**	**Vedolizumab**
Alibert et al.	KONO-S	61	22	40	32	n/a	12	37	13	7
	CONV	122	7	20	37		4	69	3	3
Holubar et al.	KONO-S	74	3	13	6	n/a	n/a	46		
	CONV	66	14	11	3			37		
Obi et al.	KONO-S	9	0	0	n/a	0	2	8	1	n/a
	CONV	9	0	1		1	2	8	0	
Tyrode et al.	KONO-S	30	4	18	n/a	15	n/a	21	5	2
	CONV	55	23	40		31		34	4	4
Kelm et al.	KONO-S	22	n/a	n/a	n/a	1	n/a	4	1	
	CONV	29				0		7	5	
Luglio et al.	KONO-S	36	3	11	n/a	8	n/a	12	n/a	2
	CONV	43	4	11		8		13		7
Shimada et al.	KONO-S	117	87	21	n/a	20	n/a	36	n/a	n/a
	CONV	98	67	25		13		14		
Kono et al.	KONO-S	69	n/a	n/a	n/a	n/a	n/a	n/a	n/a	n/a
	CONV	73								

Table S2. Previous treatment characteristics.

Author	Group	Sample	Experience	Surgeons	Emergency	Resection Site	Approach			Anastomosis					Resected Small Bowel
							Open	Laparoscopic	Conversion	Stapled	Hand-Sewn	End to Side	Side to Side	End to End	
Alibert et al.	KONO-S	61	n/a	n/a	n/a	Ileocolic	5	56	0	0	61	0	61	n/a	20(4.5)
	CONV	122					23	90	9	46	74	0	122		25(3.41)
Holubar et al.	KONO-S	74	n/a	13	4	Ileocolic	n/a	44	3	0	74	0	74	n/a	18.25(5.9)
	CONV	66		11	2			45	6	44	22	38	28		17.5(3.75)
Obi et al.	KONO-S	9	yes	2	n/a	Ileocolic	3	6	n/a	n/a	n/a	0	9	n/a	15.81
	CONV	9					0	9				1	8		13.78
Tyrode et al.	KONO-S	30	yes	n/a	n/a	Ileocolic	n/a	25	0	n/a	30	n/a	30	n/a	30(14.8)
	CONV	55						36	2		29		30		25(14)
Kelm et al.	KONO-S	22	yes	n/a	n/a	Ileocolic	10	12	1	0	22	n/a	22	n/a	n/a
	CONV	29					7	21	1	29	0		29		
Luglio et al.	KONO-S	36	n/a	n/a	n/a	Ileocolic	17	19	n/a	0	36	n/a	36	n/a	n/a
	CONV	43					21	22		43	0		43		
Shimada et al.	KONO-S	117	n/a	n/a	n/a	Mixed	n/a	n/a	n/a	n/a	117	n/a	117	0	n/a
	CONV	98									98		0	98	
Kono et al.	KONO-S	69	n/a	2	5	Mixed	n/a	n/a	n/a	n/a	69	n/a	69	0	n/a
	CONV	73		1	8						n/a		32	49	

Table S3. Surgical approach characteristics.

Variable	Estimate	SE	p
Year	0.024	0.056	0.676
Sample	-0.003	0.0026	0.341
Male Gender	0.197	1.17	0.873
Age	0.029	0.054	0.612
BMI	0.037	0.08	0.7
Follow Up	-0.004	0.01	0.75
Smoking	0.9	2.03	0.678
Previous Operation	-0.73	2.96	0.827
Perianal Disease	-1.6	3.38	0.718
Anti-TNF	-1.4	1.07	0.284
Laparoscopic Approach	-1.5	2.61	0.595
Stapled Conventional Anastomosis	2.88	1.86	0.262
Resected Bowel Length	-0.052	0.07	0.582

Table S4. Overall Morbidity Meta-Regression.

**Outcome**	**Study Type**	**Effect Estimate 95%CI**	**p**	**I^2**	**Heterogeneity p**	**Subgroup P**
Overall Complications	Prospective	0.47 [0.24, 0.89]	0.02	0%	0.63	0.24
	Retrospective	0.83 [0.41, 1.68]	0.6	56%	0.04	
CD≥III	Prospective	0.21 [0.03, 1.69]	0.14	-	-	0.27
	Retrospective	0.78 [0.28, 2.18]	0.63	8%	0.34	
Intrabdominal Abscess	Prospective	3.68 [0.15, 93.04]	0.43	-	-	0.26
	Retrospective	0.55 [0.27, 1.15]	0.11	0%	0.88	
SSI	Prospective	0.77 [0.20, 2.98]	0.71	-	-	0.16
	Retrospective	2.31 [1.17, 4.54]	0.02	0%	0.92	
Ileus	Prospective	1.20 [0.07, 19.89]	0.9	-	-	0.87
	Retrospective	0.94 [0.54, 1.66]	0.84	0%	0.76	
Bleeding	Prospective	0.16 [0.01, 3.17]	0.23	-	-	0.56
	Retrospective	0.43 [0.10, 1.91]	0.27	0%	0.77	
Leakage	Prospective	0.66 [0.07, 6.49]	0.72	-	-	0.54
	Retrospective	0.31 [0.14, 0.69]	0.004	0%	0.59	
Readmission	Prospective	0.37 [0.09, 1.49]	0.16	0%	0.72	0.36
	Retrospective	0.83 [0.29, 2.43]	0.74	61%	0.11	
Reoperation	Retrospective	0.12 [0.05, 0.27]	<0.001	0%	0.66	-
>i2	Prospective	0.46 [0.07, 2.97]	0.41	90%	0.001	0.57
	Retrospective	0.80 [0.48, 1.35]	0.41	0%	0.37	
Clinical Recurrence	Prospective	0.51 [0.18, 1.40]	0.19	36%	0.21	0.78
	Retrospective	0.35 [0.04, 3.50]	0.37	90%	0.001	
Operation Duration [minutes]	Prospective	11.49 [-0.05, 23.04]	0.05	46%	0.18	0.38
	Retrospective	-0.77 [-25.44, 23.90]	0.95	91%	<0.001	
LOS [days]	Prospective	-0.50 [-0.71, -0.30]	<0.001	0%	0.89	0.29
	Retrospective	-0.85 [-1.45, -0.24]	0.006	26%	0.24	

Table S5. Subgroup Analysis Based on Study Type

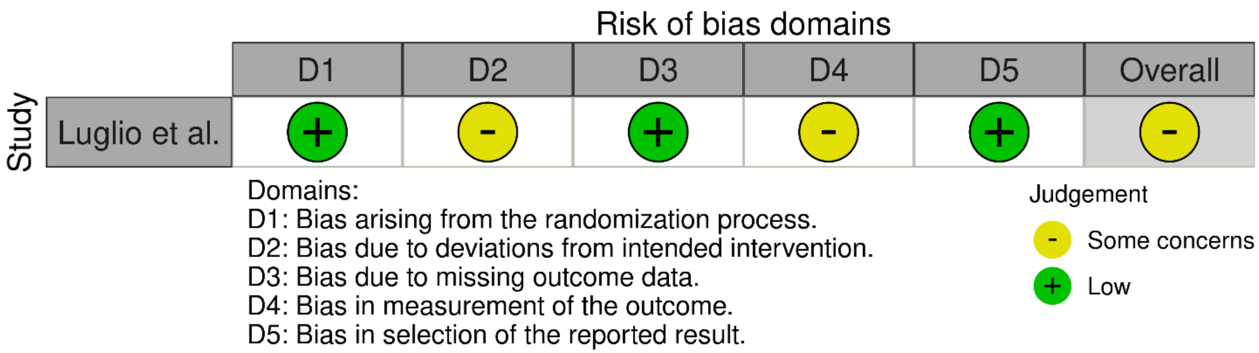

Figure S1. Risk of Bias 2 traffic light plot

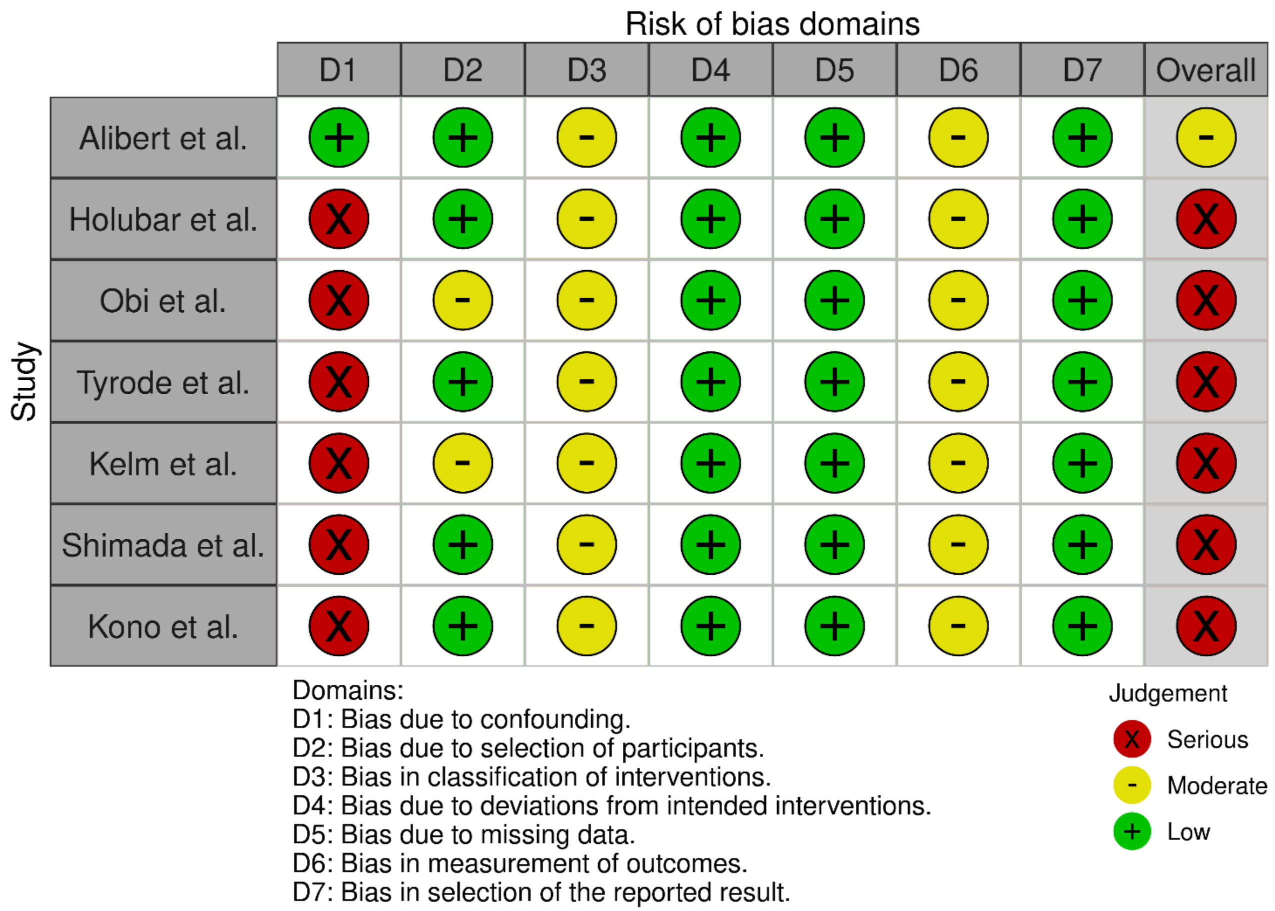

Figure S2. ROBINS-I traffic light plot

Study or Subgroup	KONO-S Events	KONO-S Total	CONV Events	CONV Total	Weight	Odds Ratio M-H, Fixed, 95% CI
Alibert et al.	1	61	9	122	41.5%	0.21 [0.03, 1.69]
Holubar et al.	2	74	5	66	36.2%	0.34 [0.06, 1.81]
Kelm et al.	4	22	3	29	14.9%	1.93 [0.38, 9.66]
Tyrode et al.	0	30	1	55	7.4%	0.60 [0.02, 15.07]
**Total (95% CI)**		**187**		**272**	**100.0%**	**0.54 [0.22, 1.32]**
Total events	7		18			

Heterogeneity: Chi² = 3.48, df = 3 (P = 0.32); I² = 14%

Test for overall effect: Z = 1.35 (P = 0.18)

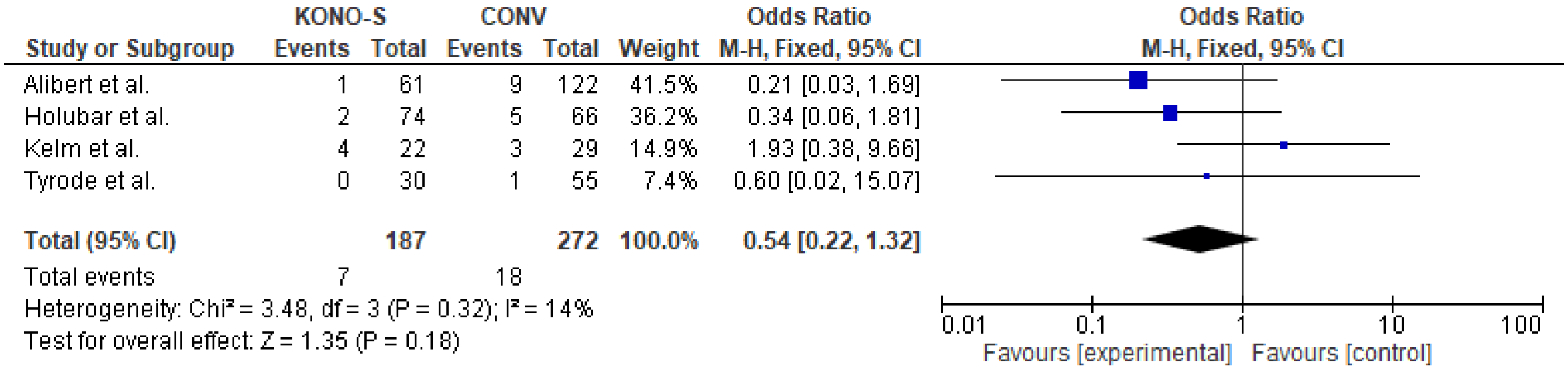

Figure S3. CD>III forest plot

Study or Subgroup	KONO-S Events	KONO-S Total	CONV Events	CONV Total	Weight	Odds Ratio M-H, Fixed, 95% CI
Holubar et al.	0	74	1	66	7.9%	0.29 [0.01, 7.32]
Kono et al.	0	69	1	73	7.3%	0.35 [0.01, 8.68]
Luglio et al.	1	36	0	43	2.2%	3.68 [0.15, 93.04]
Obi et al.	0	9	0	9		Not estimable
Shimada et al.	13	117	17	98	82.6%	0.60 [0.27, 1.30]
Tyrode et al.	0	30	55	0		Not estimable
**Total (95% CI)**		**335**		**289**	**100.0%**	**0.62 [0.31, 1.25]**
Total events	14		74			

Heterogeneity: Chi² = 1.51, df = 3 (P = 0.68); I² = 0%
Test for overall effect: Z = 1.33 (P = 0.18)

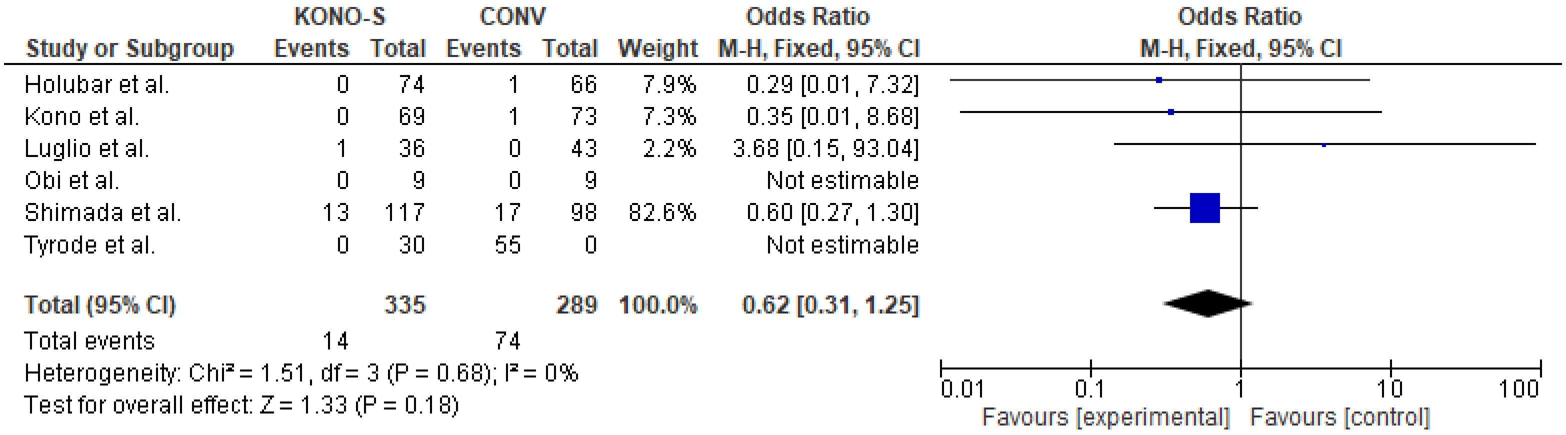

Figure S4. Intraabdominal abscess forest plot

Study or Subgroup	KONO-S Events	KONO-S Total	CONV Events	CONV Total	Weight	Odds Ratio M-H, Fixed, 95% CI
Holubar et al.	0	74	0	66		Not estimable
Kelm et al.	10	22	6	29	17.2%	3.19 [0.93, 10.92]
Kono et al.	2	69	2	73	11.5%	1.06 [0.15, 7.74]
Luglio et al.	4	36	6	43	29.7%	0.77 [0.20, 2.98]
Obi et al.	1	9	0	9	2.6%	3.35 [0.12, 93.83]
Shimada et al.	15	117	6	98	34.8%	2.25 [0.84, 6.06]
Tyrode et al.	1	30	1	55	4.2%	1.86 [0.11, 30.88]
**Total (95% CI)**		**357**		**373**	**100.0%**	**1.85 [1.02, 3.35]**
Total events	33		21			

Heterogeneity: Chi² = 2.95, df = 5 (P = 0.71); I² = 0%

Test for overall effect: Z = 2.04 (P = 0.04)

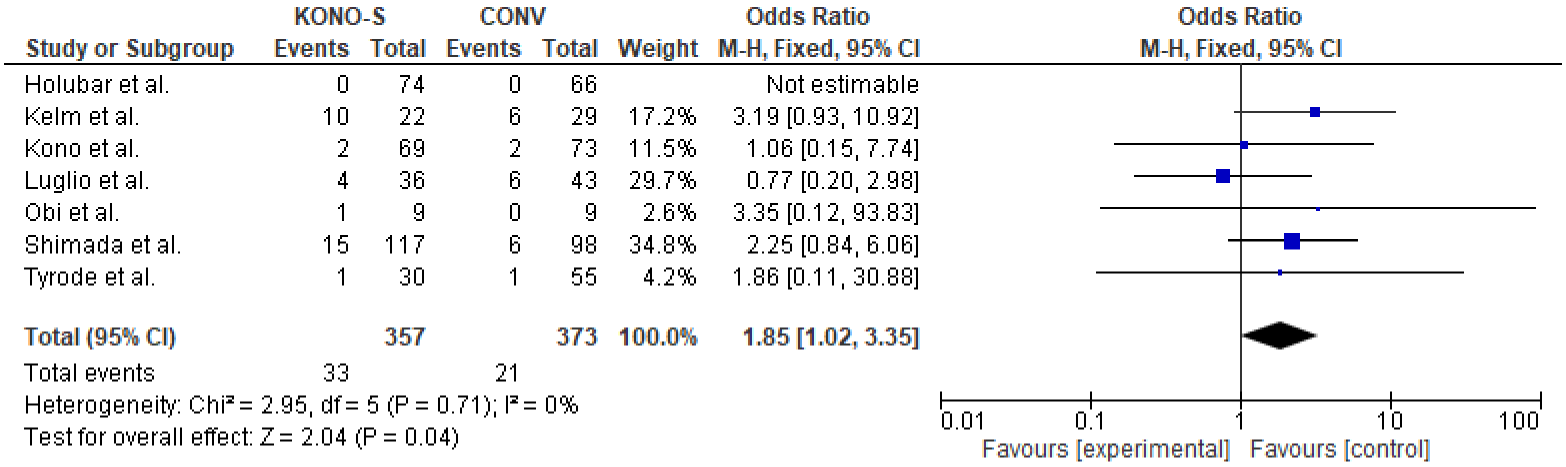

Figure S5. SSI forest plot

Study or Subgroup	KONO-S Events	KONO-S Total	CONV Events	CONV Total	Weight	Odds Ratio M-H, Fixed, 95% CI
Holubar et al.	10	74	12	66	42.9%	0.70 [0.28, 1.75]
Kelm et al.	4	22	3	29	8.3%	1.93 [0.38, 9.66]
Kono et al.	1	69	1	73	3.7%	1.06 [0.06, 17.27]
Luglio et al.	1	36	1	43	3.5%	1.20 [0.07, 19.89]
Obi et al.	0	9	0	9		Not estimable
Shimada et al.	13	117	11	98	41.6%	0.99 [0.42, 2.32]
**Total (95% CI)**		**327**		**318**	**100.0%**	**0.95 [0.55, 1.66]**
Total events	29		28			

Heterogeneity: Chi² = 1.19, df = 4 (P = 0.88); I² = 0%

Test for overall effect: Z = 0.17 (P = 0.87)

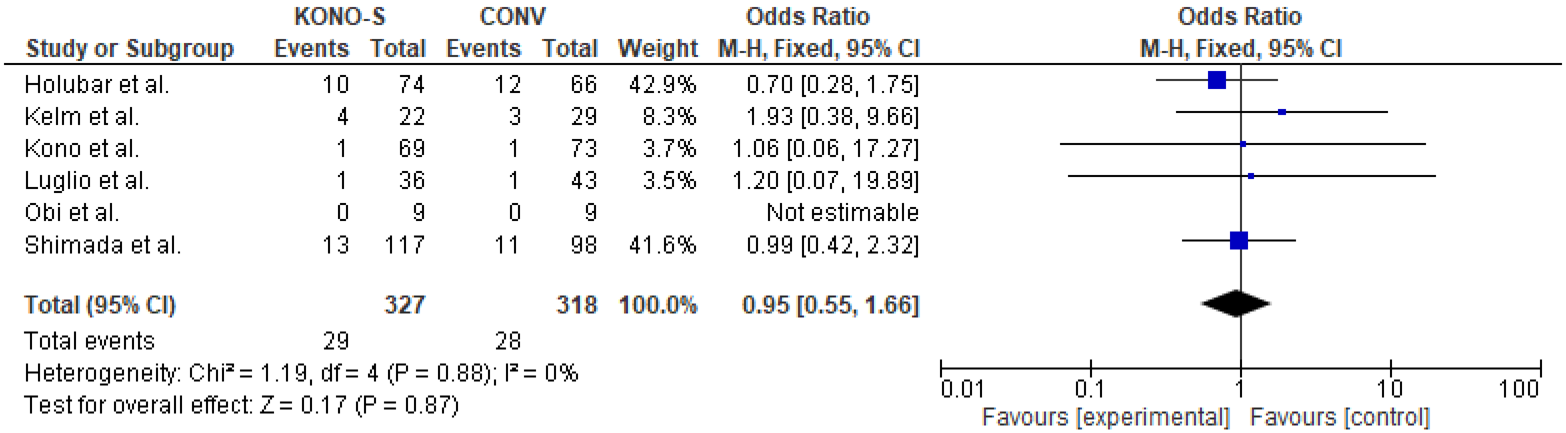

Figure S6. Ileus forest plot

Study or Subgroup	KONO-S Events	KONO-S Total	CONV Events	CONV Total	Weight	Odds Ratio M-H, Fixed, 95% CI
Holubar et al.	1	74	3	66	34.8%	0.29 [0.03, 2.84]
Kono et al.	1	69	1	73	10.6%	1.06 [0.06, 17.27]
Luglio et al.	0	36	3	43	35.1%	0.16 [0.01, 3.17]
Tyrode et al.	0	30	2	55	19.5%	0.35 [0.02, 7.55]
**Total (95% CI)**		**209**		**237**	**100.0%**	**0.34 [0.09, 1.25]**
Total events	2		9			

Heterogeneity: Chi² = 0.91, df = 3 (P = 0.82); I² = 0%

Test for overall effect: Z = 1.63 (P = 0.10)

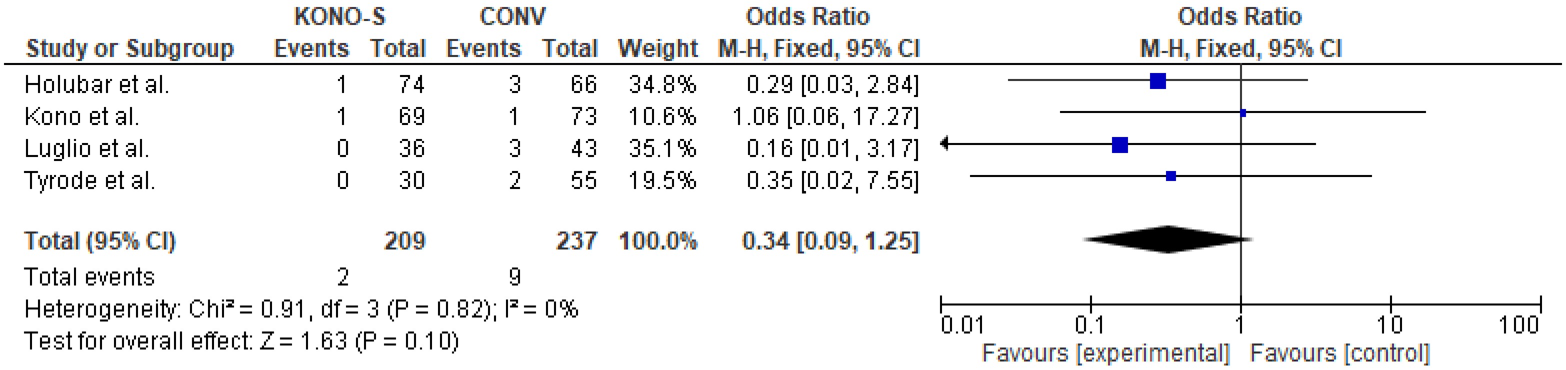

Figure S7 . Bleeding forest plot

Study or Subgroup	KONO-S Events	KONO-S Total	CONV Events	CONV Total	Weight	Odds Ratio M-H, Fixed, 95% CI
Alibert et al.	1	61	3	122	7.5%	0.66 [0.07, 6.49]
Holubar et al.	0	74	1	66	6.0%	0.29 [0.01, 7.32]
Kelm et al.	1	22	0	29	1.5%	4.12 [0.16, 106.00]
Kono et al.	0	69	3	73	12.8%	0.14 [0.01, 2.86]
Luglio et al.	0	36	0	43		Not estimable
Obi et al.	0	9	1	9	5.4%	0.30 [0.01, 8.35]
Shimada et al.	6	117	17	98	66.7%	0.26 [0.10, 0.68]
**Total (95% CI)**		**388**		**440**	**100.0%**	**0.34 [0.16, 0.71]**
Total events	8		25			

Heterogeneity: Chi² = 3.23, df = 5 (P = 0.66); I² = 0%

Test for overall effect: Z = 2.85 (P = 0.004)

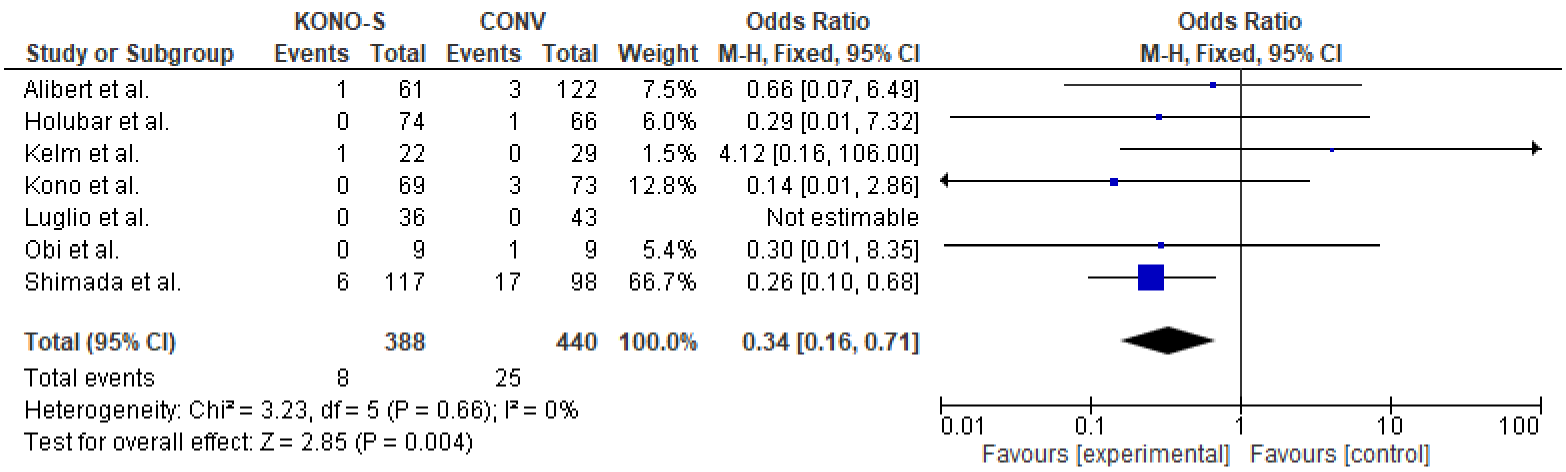

Figure S8. Leakage forest plot

Study or Subgroup	KONO-S Events	KONO-S Total	CONV Events	CONV Total	Weight	Odds Ratio M-H, Fixed, 95% CI
Alibert et al.	2	61	9	122	37.6%	0.43 [0.09, 2.03]
Holubar et al.	4	74	7	66	45.3%	0.48 [0.13, 1.73]
Kelm et al.	2	22	0	29	2.5%	7.20 [0.33, 157.85]
Luglio et al.	0	36	2	43	14.6%	0.23 [0.01, 4.89]
**Total (95% CI)**		**193**		**260**	**100.0%**	**0.59 [0.26, 1.35]**
Total events	8		18			

Heterogeneity: Chi² = 3.16, df = 3 (P = 0.37); I² = 5%

Test for overall effect: Z = 1.24 (P = 0.21)

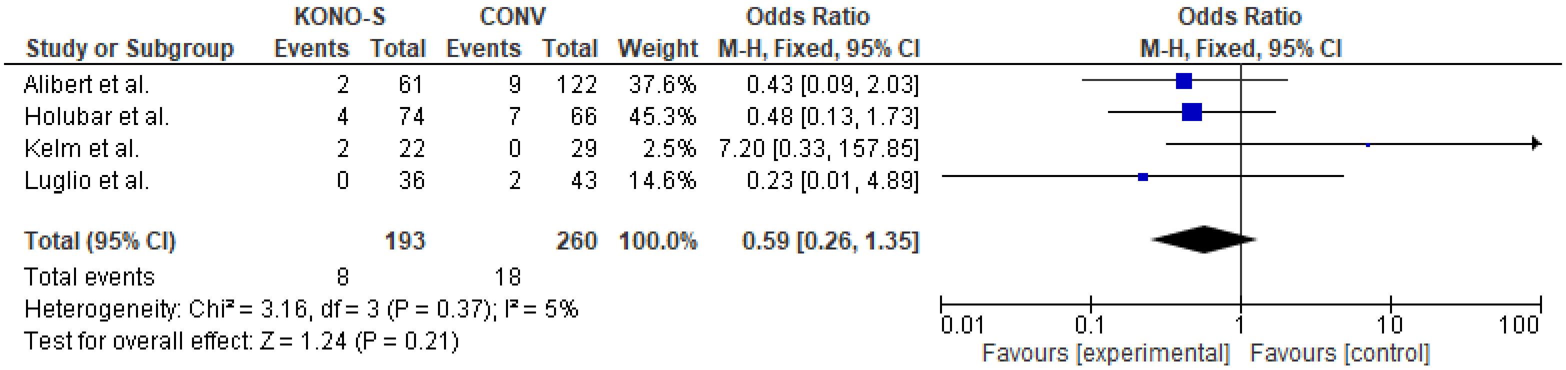

Figure S9. Readmission forest plot

Study or Subgroup	KONO-S Events	KONO-S Total	CONV Events	CONV Total	Weight	Odds Ratio M-H, Fixed, 95% CI
Holubar et al.	1	74	2	66	4.5%	0.44 [0.04, 4.95]
Kono et al.	2	69	19	73	38.4%	0.08 [0.02, 0.38]
Obi et al.	0	9	1	9	3.1%	0.30 [0.01, 8.35]
Shimada et al.	4	117	24	98	54.1%	0.11 [0.04, 0.33]
**Total (95% CI)**		**269**		**246**	**100.0%**	**0.12 [0.05, 0.27]**
Total events	7		46			

Heterogeneity: Chi² = 1.62, df = 3 (P = 0.66); I² = 0%

Test for overall effect: Z = 5.22 (P < 0.00001)

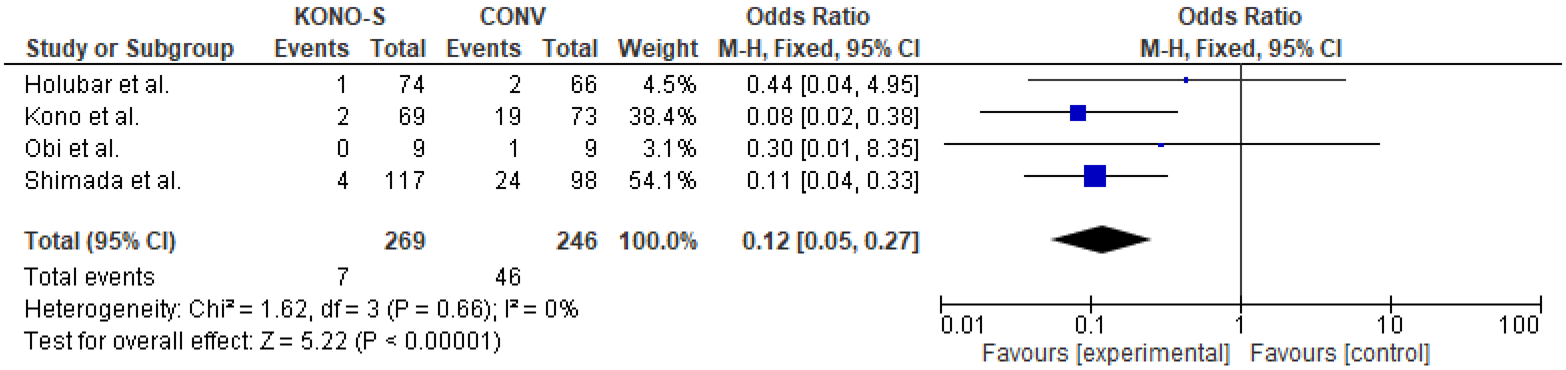

Figure S10. Reoperation forest plot

Study or Subgroup	KONO-S Events	KONO-S Total	CONV Events	CONV Total	Weight	Odds Ratio M-H, Random, 95% CI
Alibert et al.	29	61	54	122	24.2%	1.14 [0.62, 2.11]
Kelm et al.	7	22	13	29	16.0%	0.57 [0.18, 1.83]
Kono et al.	49	69	58	73	21.8%	0.63 [0.29, 1.37]
Luglio et al.	8	36	27	43	18.2%	0.17 [0.06, 0.46]
Tyrode et al.	17	30	27	55	19.8%	1.36 [0.55, 3.32]
**Total (95% CI)**		**218**		**322**	**100.0%**	**0.66 [0.33, 1.29]**
Total events	110		179			

Heterogeneity: Tau² = 0.39; Chi² = 12.37, df = 4 (P = 0.01); I² = 68%
Test for overall effect: Z = 1.22 (P = 0.22)

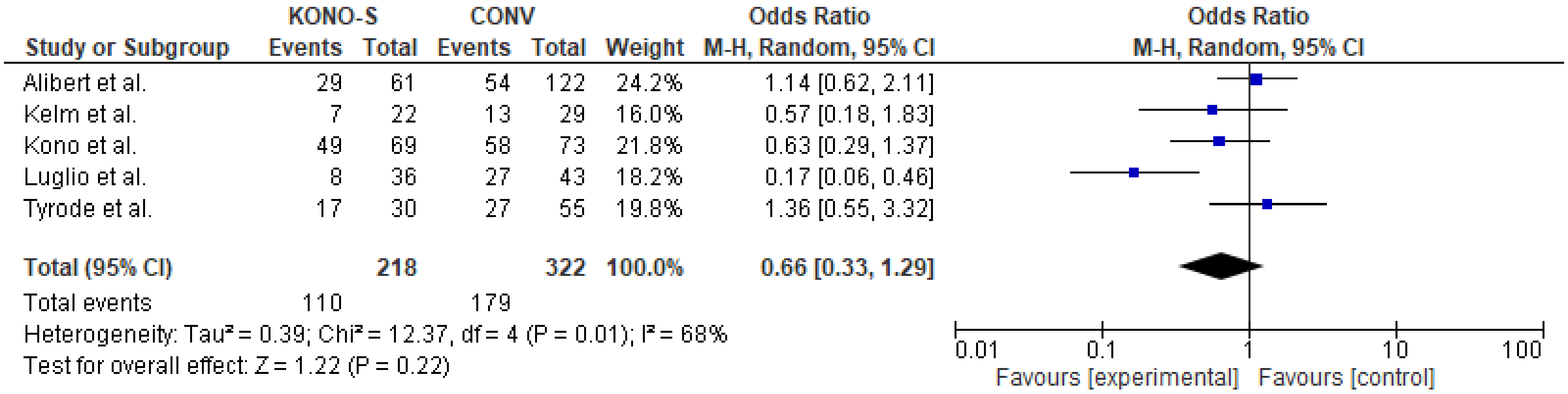

Figure S11. >i2 forest plot

Study or Subgroup	KONO-S Events	KONO-S Total	CONV Events	CONV Total	Weight	Odds Ratio M-H, Random, 95% CI
Alibert et al.	5	61	12	122	24.8%	0.82 [0.27, 2.44]
Luglio et al.	4	36	13	43	23.3%	0.29 [0.08, 0.98]
Shimada et al.	4	117	24	98	24.8%	0.11 [0.04, 0.33]
Tyrode et al.	16	30	28	55	27.2%	1.10 [0.45, 2.69]
**Total (95% CI)**		**244**		**318**	**100.0%**	**0.42 [0.14, 1.24]**
Total events	29		77			

Heterogeneity: $Tau^2 = 0.90$; $Chi^2 = 12.08$, df = 3 (P = 0.007); $I^2 = 75\%$
Test for overall effect: Z = 1.57 (P = 0.12)

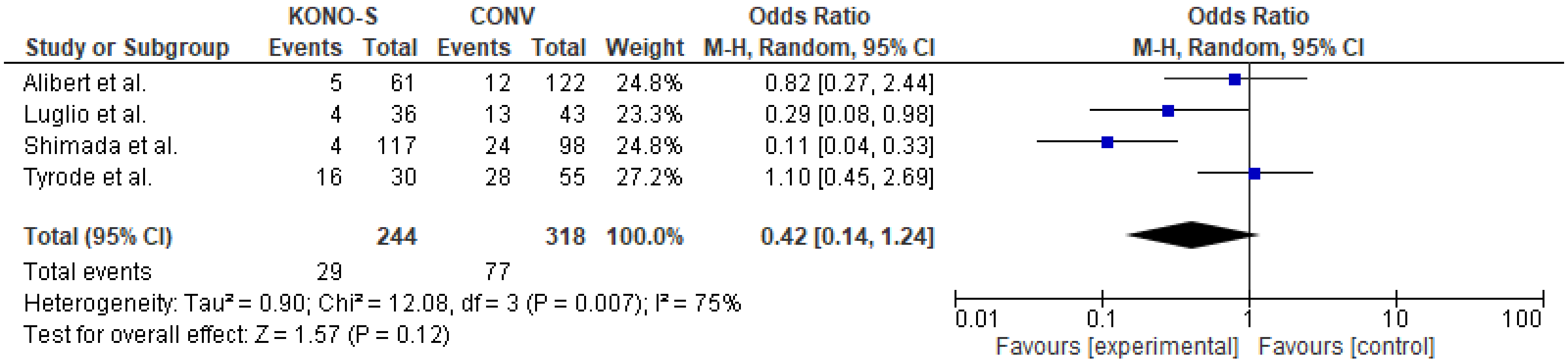

Figure S12. Clinical recurrence forest plot

	KONO-S			CONV				Mean Difference	Mean Difference
Study or Subgroup	Mean [minutes]	SD [minutes]	Total	Mean [minutes]	SD [minutes]	Total	Weight	IV, Random, 95% CI	IV, Random, 95% CI
Alibert et al.	140	18.5	61	125	7.33	122	26.8%	15.00 [10.18, 19.82]	
Holubar et al.	170	17.45	74	150	17.8	66	26.1%	20.00 [14.15, 25.85]	
Luglio et al.	165	42	36	163.4	42.8	43	15.1%	1.60 [-17.16, 20.36]	
Shimada et al.	215	71.85	117	239	88.8	98	12.9%	-24.00 [-45.88, -2.12]	
Tyrode et al.	94.5	27.4	30	98	37	55	19.1%	-3.50 [-17.35, 10.35]	
**Total (95% CI)**			**318**			**384**	**100.0%**	**5.71 [-4.93, 16.36]**	

Heterogeneity: Tau² = 104.00; Chi² = 23.60, df = 4 (P < 0.0001); I² = 83%
Test for overall effect: Z = 1.05 (P = 0.29)

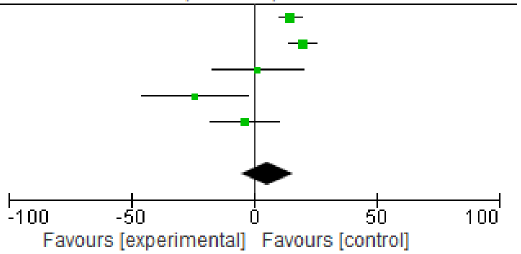

Figure S13. Operation duration forest plot

	KONO-S			CONV				Mean Difference
**Study or Subgroup**	**Mean [days]**	**SD [days]**	**Total**	**Mean [days]**	**SD [days]**	**Total**	**Weight**	**IV, Fixed, 95% CI**
Alibert et al.	6	0.75	61	6.5	0.5	122	87.6%	-0.50 [-0.71, -0.29]
Holubar et al.	4	4.25	74	4	5	66	1.6%	0.00 [-1.55, 1.55]
Luglio et al.	7	3	36	7.6	3.08	43	2.1%	-0.60 [-1.94, 0.74]
Tyrode et al.	6	1.48	30	7	1.48	55	8.7%	-1.00 [-1.66, -0.34]
**Total (95% CI)**			**201**			**286**	**100.0%**	**-0.54 [-0.73, -0.34]**

Heterogeneity: Chi² = 2.49, df = 3 (P = 0.48); I² = 0%
Test for overall effect: Z = 5.41 (P < 0.00001)

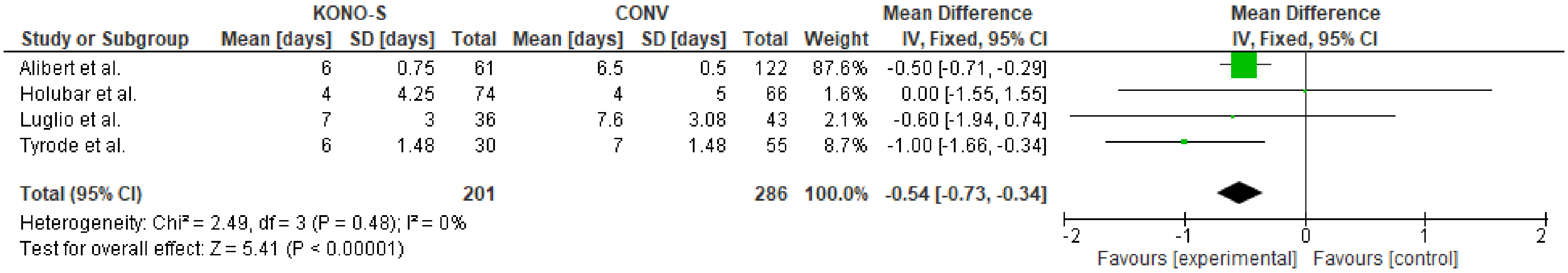

Figure S14. LOS forest plot

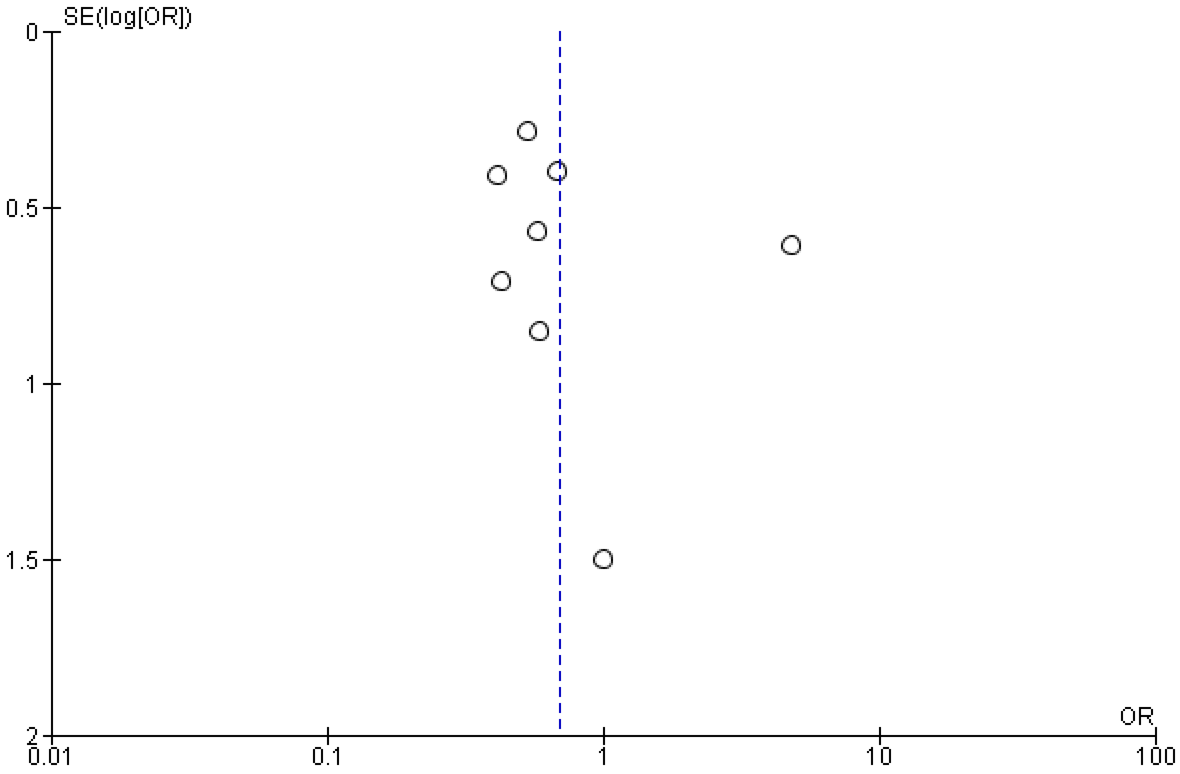

Figure S15. Overall complications funnel plot

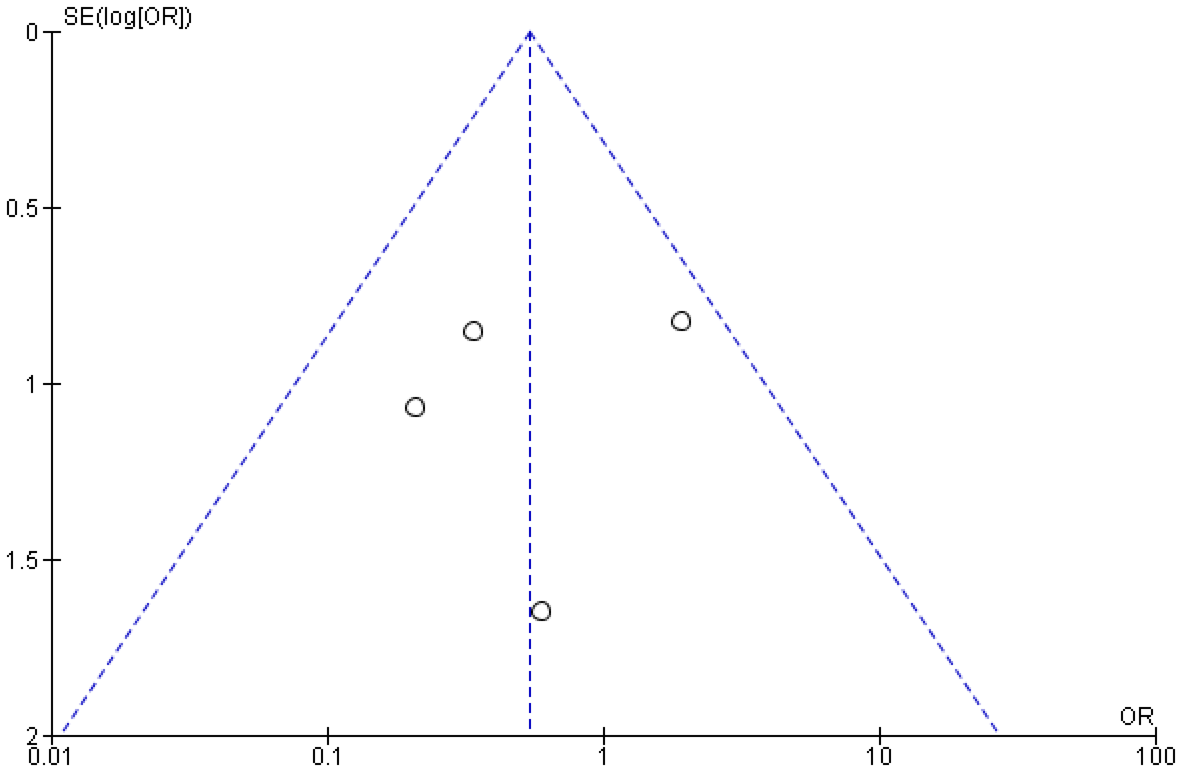

Figure S16. CD>III funnel plot

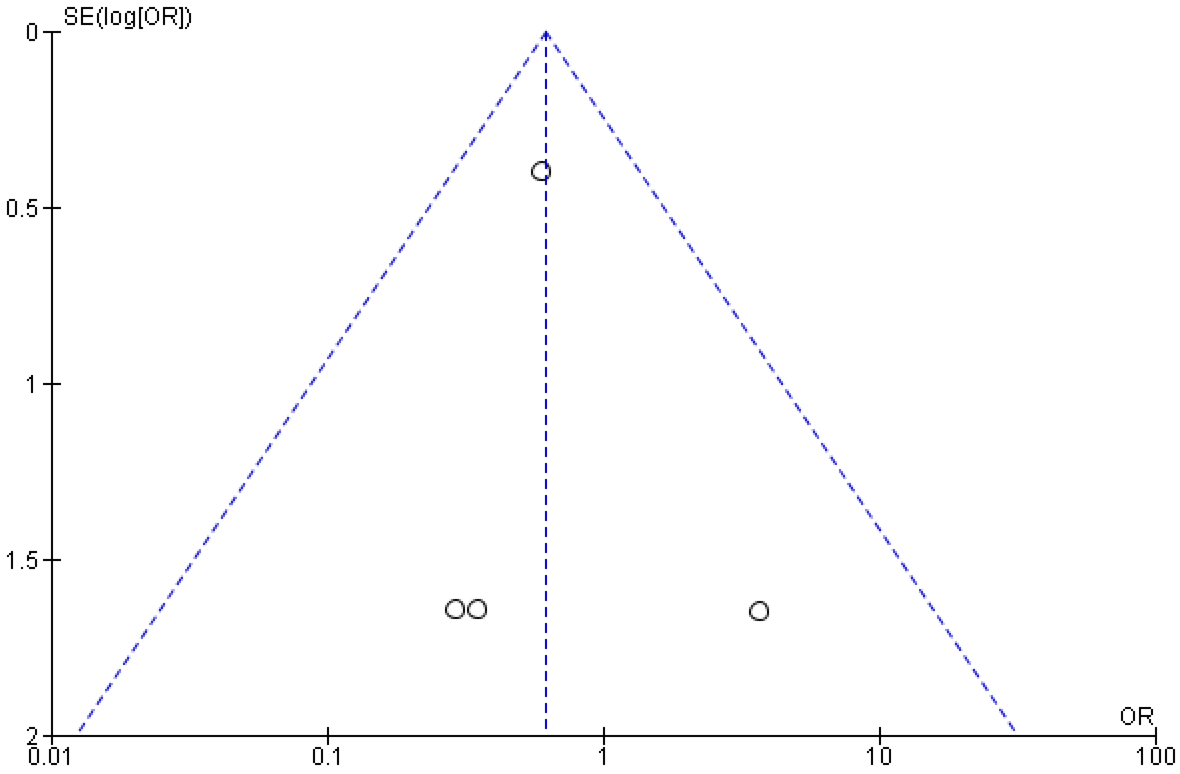

Figure S17. Intraabdominal abscess funnel plot

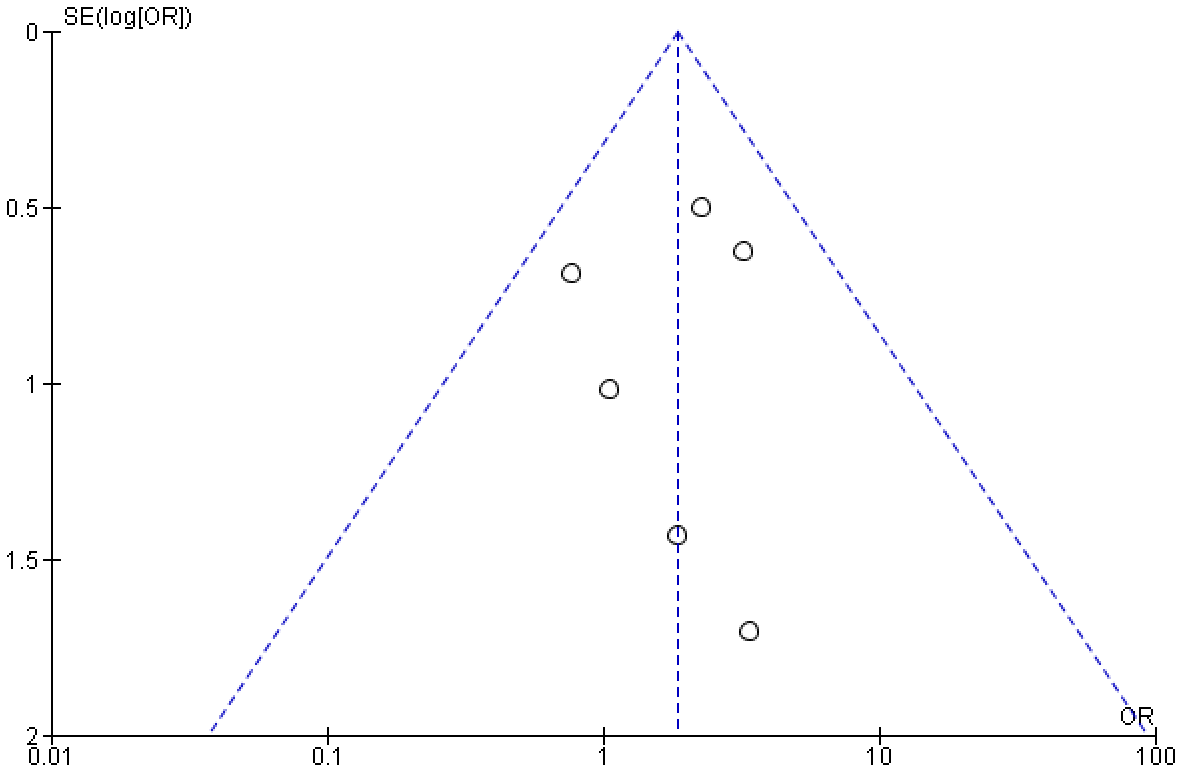

Figure S18. SSI funnel plot

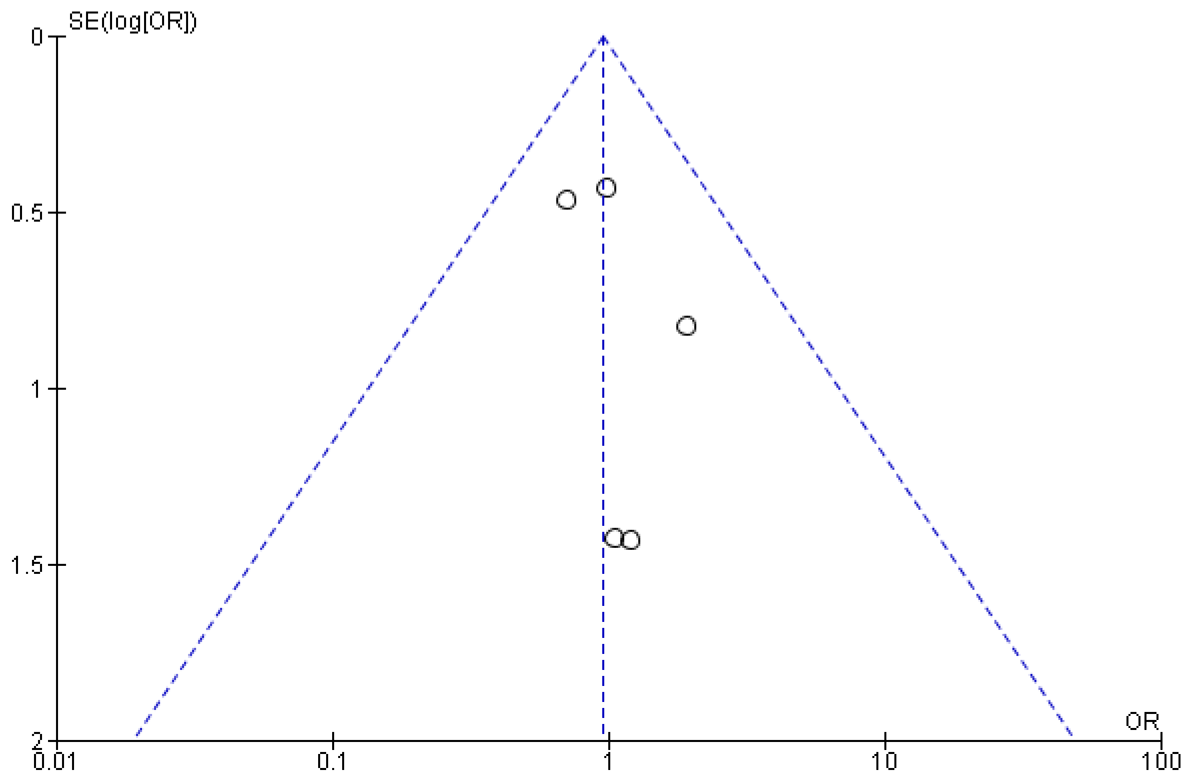

Figure S19. Ileus funnel plot

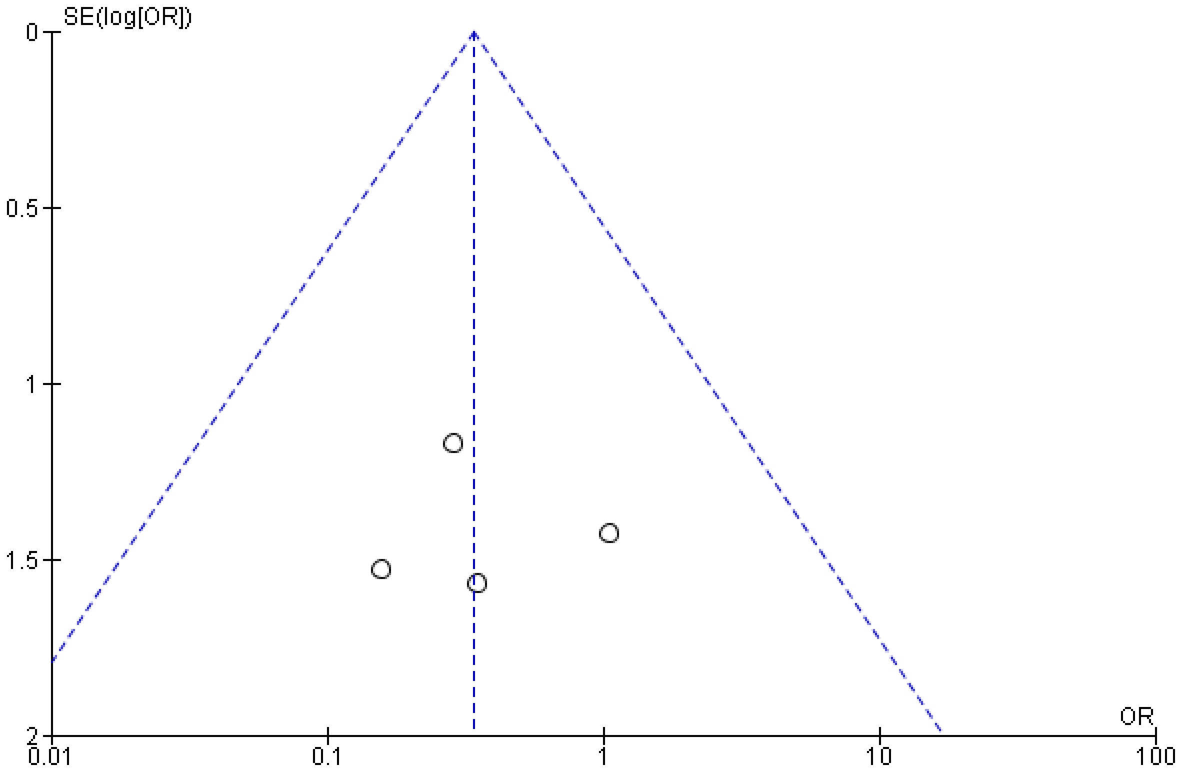

Figure S20. Bleeding funnel plot

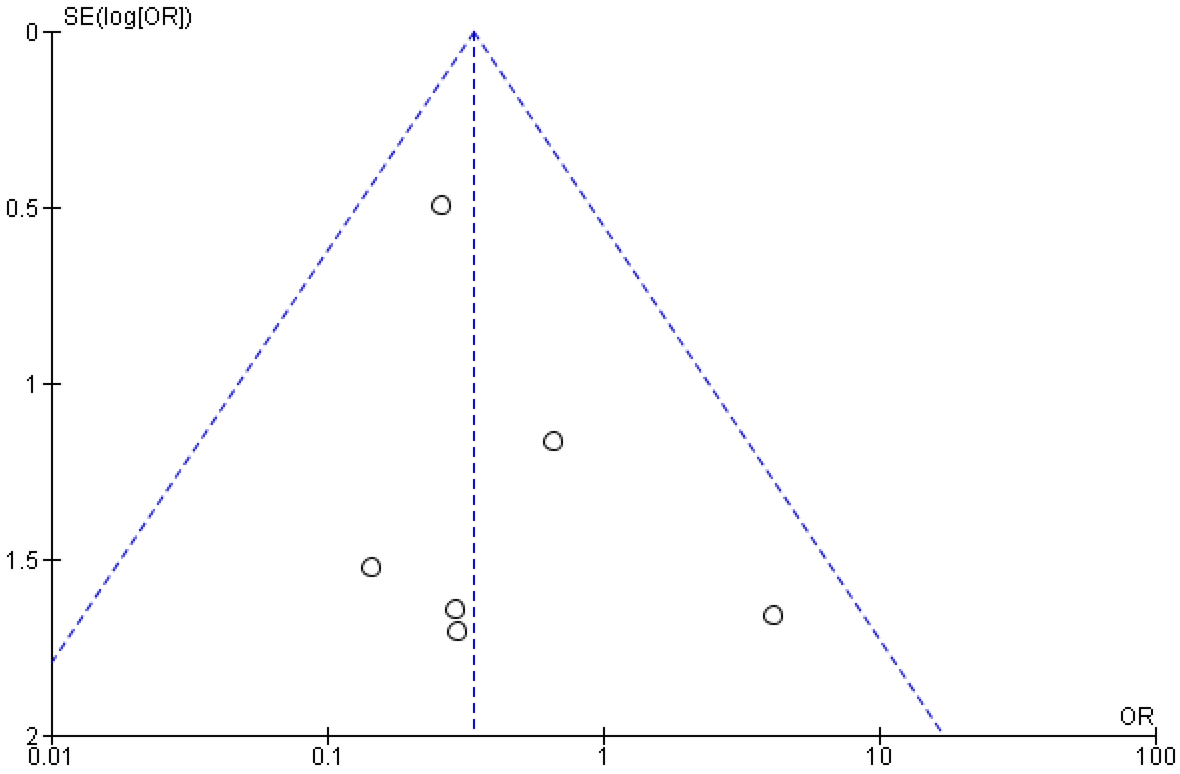

Figure S21. Leakage funnel plot

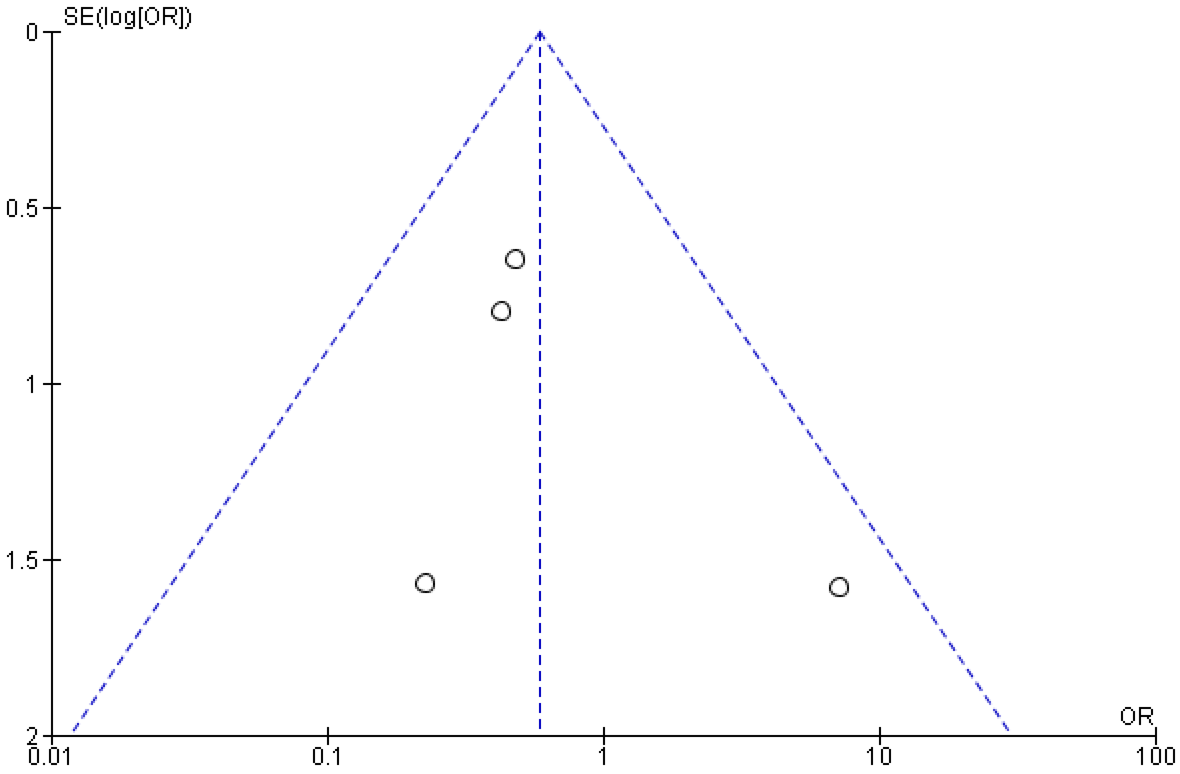

Figure S22. Readmission funnel plot

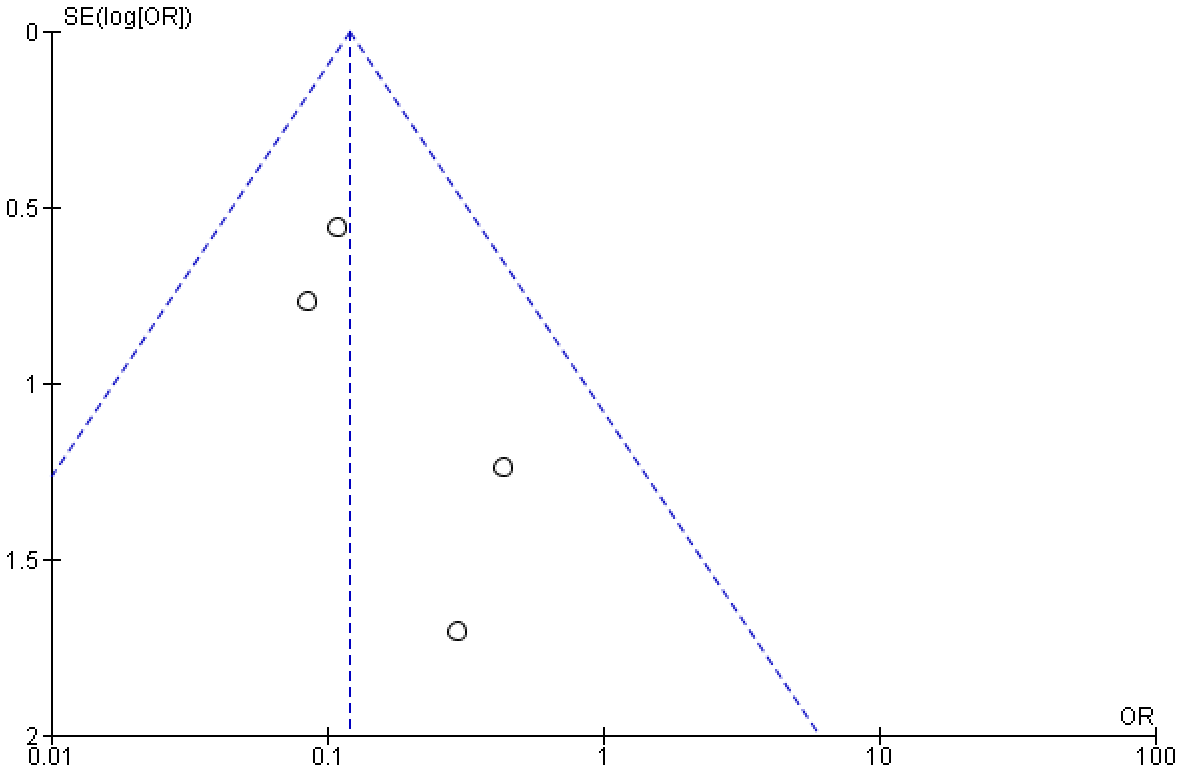

Figure S23. Reoperation funnel plot

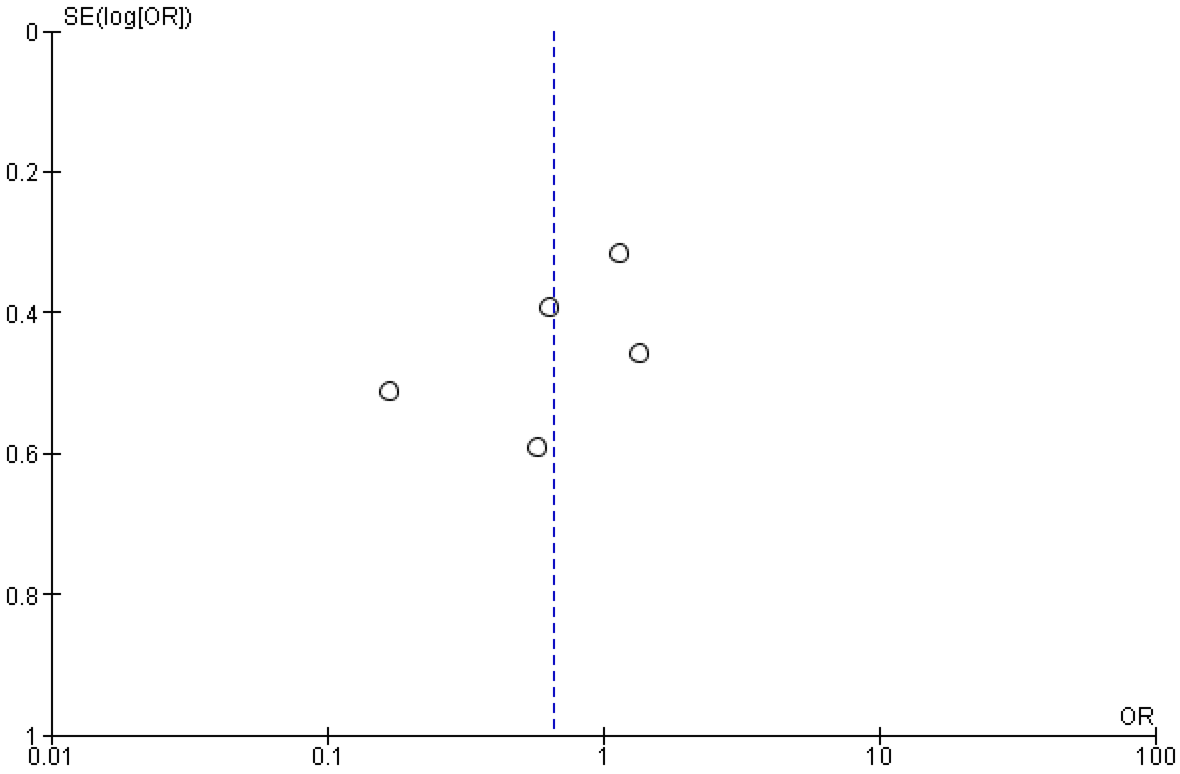

Figure S24. >i2 funnel plot

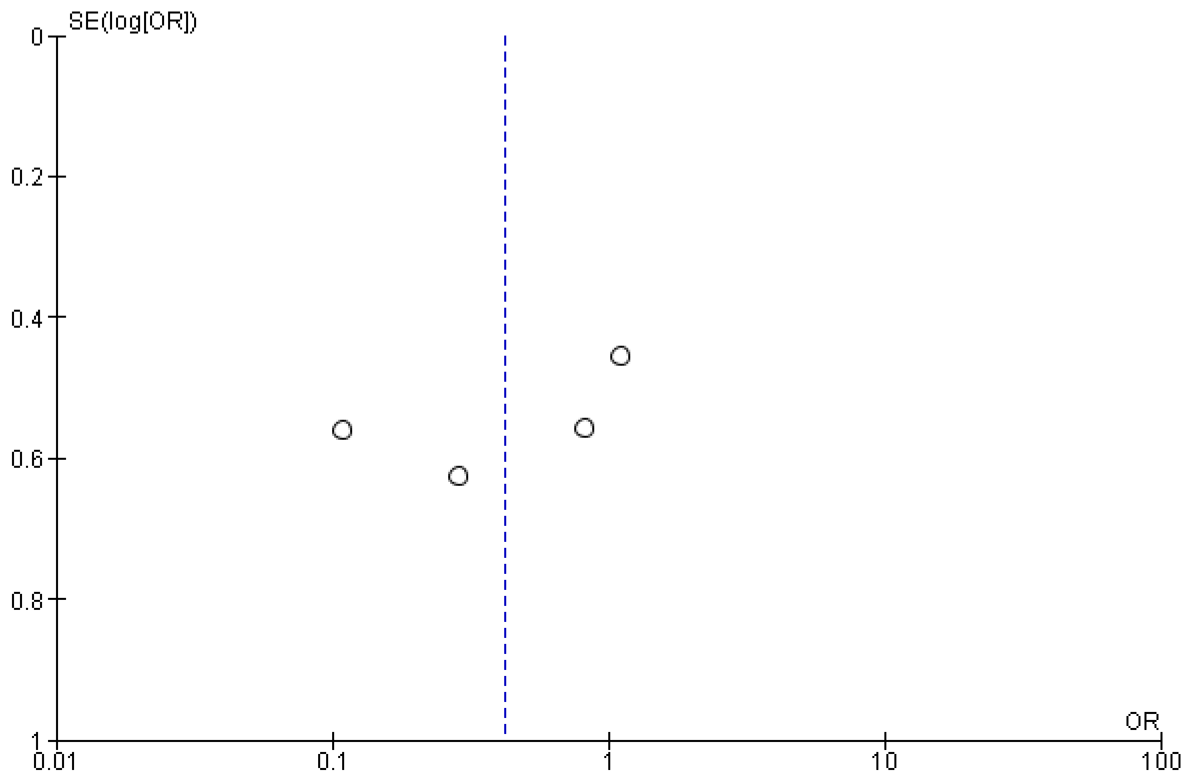

Figure S25. Clinical recurrence funnel plot

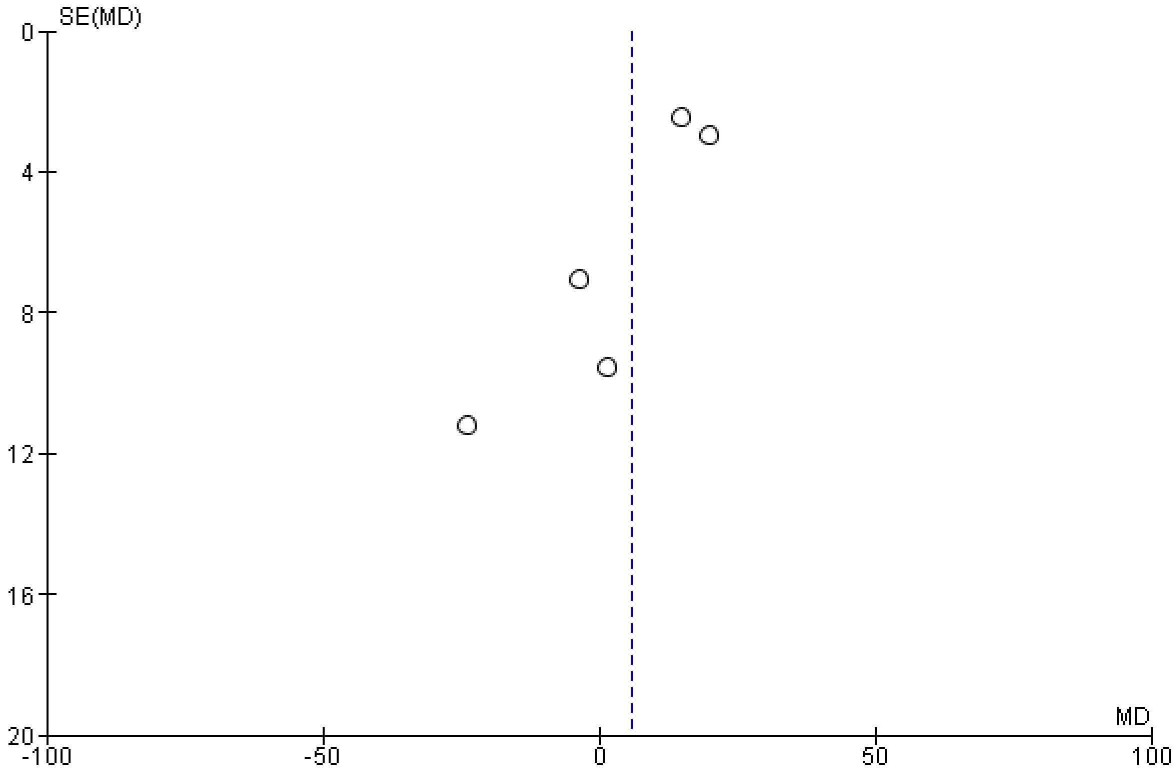

Figure S26. Operation duration funnel plot

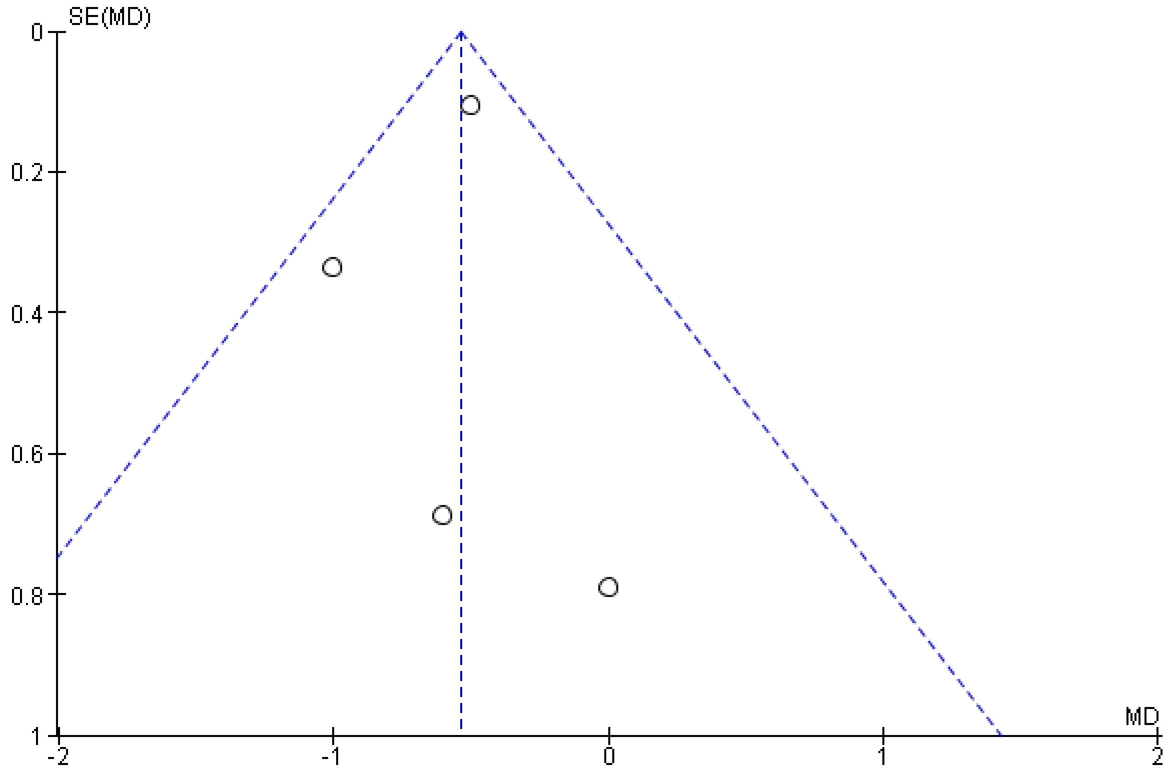

Figure S27. LOS funnel plot

Section and Topic	Item #	Checklist item	Location where item is reported
**TITLE**			
Title	1	Identify the report as a systematic review.	1
**ABSTRACT**			
Abstract	2	See the PRISMA 2020 for Abstracts checklist.	1
**INTRODUCTION**			
Rationale	3	Describe the rationale for the review in the context of existing knowledge.	1,2
Objectives	4	Provide an explicit statement of the objective(s) or question(s) the review addresses.	2
**METHODS**			
Eligibility criteria	5	Specify the inclusion and exclusion criteria for the review and how studies were grouped for the syntheses.	2
Information sources	6	Specify all databases, registers, websites, organisations, reference lists and other sources searched or consulted to identify studies. Specify the date when each source was last searched or consulted.	3
Search strategy	7	Present the full search strategies for all databases, registers and websites, including any filters and limits used.	3
Selection process	8	Specify the methods used to decide whether a study met the inclusion criteria of the review, including how many reviewers screened each record and each report retrieved, whether they worked independently, and if applicable, details of automation tools used in the process.	3
Data collection process	9	Specify the methods used to collect data from reports, including how many reviewers collected data from each report, whether they worked independently, any processes for obtaining or confirming data from study investigators, and if applicable, details of automation tools used in the process.	3
Data items	10a	List and define all outcomes for which data were sought. Specify whether all results that were compatible with each outcome domain in each study were sought (e.g. for all measures, time points, analyses), and if not, the methods used to decide which results to collect.	2,3
	10b	List and define all other variables for which data were sought (e.g. participant and intervention characteristics, funding sources). Describe any assumptions made about any missing or unclear information.	2,3
Study risk of bias assessment	11	Specify the methods used to assess risk of bias in the included studies, including details of the tool(s) used, how many reviewers assessed each study and whether they worked independently, and if applicable, details of automation tools used in the process.	3
Effect measures	12	Specify for each outcome the effect measure(s) (e.g. risk ratio, mean difference) used in the synthesis or presentation of results.	3
Synthesis methods	13a	Describe the processes used to decide which studies were eligible for each synthesis (e.g. tabulating the study intervention characteristics and comparing against the planned groups for each synthesis (item #5)).	3
	13b	Describe any methods required to prepare the data for presentation or synthesis, such as handling of missing summary	3

Section and Topic	Item #	Checklist item	Location where item is reported
		statistics, or data conversions.	
	13c	Describe any methods used to tabulate or visually display results of individual studies and syntheses.	3
	13d	Describe any methods used to synthesize results and provide a rationale for the choice(s). If meta-analysis was performed, describe the model(s), method(s) to identify the presence and extent of statistical heterogeneity, and software package(s) used.	3
	13e	Describe any methods used to explore possible causes of heterogeneity among study results (e.g. subgroup analysis, meta-regression).	3
	13f	Describe any sensitivity analyses conducted to assess robustness of the synthesized results.	3
Reporting bias assessment	14	Describe any methods used to assess risk of bias due to missing results in a synthesis (arising from reporting biases).	3
Certainty assessment	15	Describe any methods used to assess certainty (or confidence) in the body of evidence for an outcome.	n/a
**RESULTS**			
Study selection	16a	Describe the results of the search and selection process, from the number of records identified in the search to the number of studies included in the review, ideally using a flow diagram.	3, Figure 1
	16b	Cite studies that might appear to meet the inclusion criteria, but which were excluded, and explain why they were excluded.	3
Study characteristics	17	Cite each included study and present its characteristics.	3, 4 Table 1
Risk of bias in studies	18	Present assessments of risk of bias for each included study.	4
Results of individual studies	19	For all outcomes, present, for each study: (a) summary statistics for each group (where appropriate) and (b) an effect estimate and its precision (e.g. confidence/credible interval), ideally using structured tables or plots.	4, Table 2
Results of syntheses	20a	For each synthesis, briefly summarise the characteristics and risk of bias among contributing studies.	Supp. Mat,
	20b	Present results of all statistical syntheses conducted. If meta-analysis was done, present for each the summary estimate and its precision (e.g. confidence/credible interval) and measures of statistical heterogeneity. If comparing groups, describe the direction of the effect.	4, Table 2, Supp. Mat.
	20c	Present results of all investigations of possible causes of heterogeneity among study results.	4, Supp.Mat.
	20d	Present results of all sensitivity analyses conducted to assess the robustness of the synthesized results.	4,

Section and Topic	Item #	Checklist item	Location where item is reported
			Supp.Mat.
Reporting biases	21	Present assessments of risk of bias due to missing results (arising from reporting biases) for each synthesis assessed.	n/a
Certainty of evidence	22	Present assessments of certainty (or confidence) in the body of evidence for each outcome assessed.	n/a
**DISCUSSION**			
Discussion	23a	Provide a general interpretation of the results in the context of other evidence.	6-9
	23b	Discuss any limitations of the evidence included in the review.	8
	23c	Discuss any limitations of the review processes used.	8
	23d	Discuss implications of the results for practice, policy, and future research.	9
**OTHER INFORMATION**			
Registration and protocol	24a	Provide registration information for the review, including register name and registration number, or state that the review was not registered.	n/a
	24b	Indicate where the review protocol can be accessed, or state that a protocol was not prepared.	n/a
	24c	Describe and explain any amendments to information provided at registration or in the protocol.	n/a
Support	25	Describe sources of financial or non-financial support for the review, and the role of the funders or sponsors in the review.	9
Competing interests	26	Declare any competing interests of review authors.	9
Availability of data, code and other materials	27	Report which of the following are publicly available and where they can be found: template data collection forms; data extracted from included studies; data used for all analyses; analytic code; any other materials used in the review.	9

From: Page MJ, McKenzie JE, Bossuyt PM, Boutron I, Hoffmann TC, Mulrow CD, et al. The PRISMA 2020 statement: an updated guideline for reporting systematic reviews. BMJ 2021;372:n71. doi: 10.1136/bmj.n71

For more information, visit: http://www.prisma-statement.org/
